# Using ngTALEN to improve genome editing efficiency on targets containing 5‐methylcytosines

**DOI:** 10.1111/tpj.70826

**Published:** 2026-04-04

**Authors:** Zhicai Wang, Yoshiko Tamura, Masaru Hashimoto, Issei Nakazato, Asuka Yamaguchi‐Nishimura, Shin‐ichi Arimura

**Affiliations:** ^1^ Laboratory of Plant Molecular Genetics, Graduate School of Agricultural and Life Sciences The University of Tokyo 1‐1‐1 Yayoi Bunkyo‐ku Tokyo 113‐8657 Japan; ^2^ Key Laboratory of National Forestry and Grassland Administration for Orchid Conservation and Utilization The National Orchid Conservation Center of China and the Orchid Conservation & Research Center of Shenzhen 889 Wangtong Rd. Luohu Shenzhen 518114 China

**Keywords:** genome editing, ngTALEN, 5‐Methylcytosine, *Arabidopsis thaliana*, *Flowering Wageningen*

## Abstract

We recently discovered distinct methylation patterns between the mitochondrial genome and the nuclear‐encoded mitochondrial DNA sequences (NUMTs), with the mitochondrial genome being hypomethylated and NUMTs being hypermethylated. Given that genome editing using mitochondrial targeted transcription activator‐like effector nucleases (TALEN) is highly efficient, while editing at NUMT is difficult, we hypothesized that the methylation status might affect editing outcomes. To test this, we attempted to use ngTALEN [employing RVD‐NG to recognize 5‐methylcytosine (5mC)] to target the *Flowering Wageningen* (*FWA*) locus of *Arabidopsis thaliana*, specifically the promoter and gene body regions with varying levels of cytosine methylation. Comparative analysis using the active epimutant allele *fwa‐d* and wild‐type Columbia‐0 (Col‐0) carrying a naturally silenced allele of *FWA* revealed that editing was impeded by 5mC at both the promoter and gene body of *FWA* for both CRISPR/Cas9 and TALEN. Overall, TALEN editing is robust and comparable to that of CRISPR/Cas9 at multiple sites, while ngTALEN showed improved editing at the CG‐hypermethylated promoter of *FWA* compared with TALEN. Additionally, when targeting multiple genomic loci with identical sequences that differ in methylation levels and chromatin states, ngTALEN was less effective to induce edits. Therefore, this study represents the first systematic comparison of editing efficiency between CRISPR/Cas9 and TALEN in dealing with methylated or unmethylated DNA in plants. Furthermore, we have developed ngTALEN as a specific and robust tool for enhancing editing at sites with various levels of CG methylation.

## Significance Statement

To overcome the inhibition of editing caused by methylation, we utilized ngTALEN, which has demonstrated a strong binding affinity to 5‐methylcytosine (5mC) through *in vitro* chemical binding assays, to recognize 5mC at methylated sites in plants. By systematic comparing the effectiveness of three editors, CRISPR/Cas9, TALEN, and ngTALEN, in editing overlapping windows at *FWA* locus with different 5mC statuses in both Col‐0 and *fwa* epimutant backgrounds, we reported efficient editing by ngTALEN on methylated DNA.

## INTRODUCTION

In higher eukaryotes, DNA methylation is a prominent epigenetic modification crucial for various biological functions, including gene expression and developmental regulation (He et al., 2022). Generally, DNA methylation involves the addition of a methyl group to cytosine, resulting in the formation of 5‐methylcytosine (5mC) in DNA (Choi et al., 2021). It is the most abundant and extensively studied epigenetic mark, shown to be essential for regulating gene expression without altering the DNA sequence itself (Rauluseviciute et al., 2019). In plants, this epigenetic modification occurs at CG, CHG, and CHH sequences, where H can represent any of the bases C, T, or A (Bond & Baulcombe, 2015). Recently, there has been a surge in technologies utilizing sequence‐specific nucleases (SSNs) for genome editing. Among these, CRISPR/Cas9 and transcription activator‐like effector nuclease (TALEN) are the two methods that have been widely and successfully adopted, continuing to revolutionize the field (Bhardwaj & Nain, 2021; Liu et al., 2025). Realizing the full potential of these editing technologies requires efficient access to target sites within chromatin. However, chromatin modifications and DNA methylation are prevalent across the genome, often impeding the accessibility and editing of SSNs (Daer et al., 2017).

Since 2012, CRISPR/Cas9 and many related CRISPR systems have become the world's most successful genome editing tools due to their simplicity in use and extraordinary versatility (Becker & Boch, 2021). Increasing evidence reveals that DNA sequence is the primary determinant for CRISPR mutagenesis efficiencies and mutation profiles, while DNA methylation and chromatin features also play significant roles. Studies have reported that low CRISPR/Cas9 editing frequencies are usually associated with repressive heterochromatic signatures, such as limited chromatin accessibility and histone modifications (Weiss et al., 2022). However, most of these studies examined multiple genomic loci with various target sequences and chromatin features, making it difficult to determine their respective contributions to editing frequency. To address this issue, recent studies have investigated the effect of chromatin features on editing efficiency by first randomly insertion reporters at various genomic locations and then targeting an identical sequence within the reporter for editing (Přibylová et al., 2022; Weiss et al., 2022). However, it is possible that the insertion of reporters may be biased and may not accurately reflect the local chromatin context. Another approach has been to target CRISPR/Cas9 to a specific sequence in multiple chromosomal regions with distinct DNA methylation and highly diverse chromatin contexts (Weiss et al., 2022). This study confirmed previous observations that heterochromatin inhibits CRISPR/Cas9 editing efficiency. Nevertheless, there are still too many variables related to chromatin features to clearly define which feature is the major factor contributing to editing frequencies and mutation outcomes.

TALEN is the first genome editing technology that can be designed and constructed with relative ease. It is widely used in multiple species and is capable of targeting any specific genomic locus with unmatched flexibility and precise positioning (Bhardwaj & Nain, 2021). It consists of a protein‐only system that employs paired molecules to recognize 13–20 nucleobases flanking both sides of a target site where a DNA double‐strand break (DSB) is intended to occur (Arimura, 2022). The paired TALEN binding molecules are composed of a variable number of 33–35 amino acid long tandem repeats. These repeats contain divergent 12th and 13th amino acids (repeat variable diresidues, RVDs) that determine the DNA‐binding specificity and designer capacity. Each RVD in the repeats recognizes one nucleotide in the binding sequences (Becker & Boch, 2021). Some RVDs can discriminate methylation states of nucleotides at single‐nucleotide resolution, thereby providing a wide array of tools for methylation‐dependent genome manipulation. For example, the naturally occurring RVD HD recognizes cytosine but is unable to recognize 5mC. Conversely, the RVD N* (which lacks the 13th amino acid) recognizes both methylated and non‐methylated cytosines (Becker & Boch, 2021). However, the binding affinity of N* to 5mC is not optimal (Zhang et al., 2017). Another study in rice revealed that N*‐TALEN improved genome editing efficiency at one stably methylated target but showed no activity at another target carrying cytosines with various levels of methylation (Kaya et al., 2017). In contrast, a study utilizing *in vitro* biochemical binding assays demonstrated that 5mC was bound by TALEN recognition module NG (ngTALEN) with high affinity and specificity, rather than by HD (Deng et al., 2012). Even when six 5mC bases were present in the binding site, they could still be recognized by ngTALEN, and the binding was specific and strong. Unfortunately, it has not yet been tested in plants for editing methylated DNA.

Examination of multiple sites for editing within a gene is expected to minimize differences due to factors other than cytosine methylation, thereby providing an accurate estimation of editing in methylated sites. Tandem repeat sequences are prevalent in methylated regions of the genome and are often associated with gene silencing (Chan et al., 2006). The promoter of *Arabidopsis thaliana FLOWERING WAGENINGEN* (*AtFWA*) gene contains two tandem repeats and numerous cytosines, particularly of the CG type, that are heavily methylated. Although *FWA* undergoes demethylation and expression in the gametophyte and the terminally differentiating endosperm, it remains heritably and stably methylated and silenced throughout the remainder of the plant's life cycle. A rare unmethylated epimutant of *FWA*, termed *fwa*, exhibits no change in nucleotide sequence but ectopically overexpresses *FWA* in adult tissues, causing a dominant late‐flowering phenotype (Choi et al., 2021). This unmethylated epiallele is extremely stable, and reversal of the methylation state, and hence a return to early flowering, has never been observed (Chan et al., 2006).

We recently discovered that the mitochondrial genome was hypomethylated, whereas the nuclear‐encoded mitochondrial DNA sequences (NUMTs) were hypermethylated (Zhong et al., 2025). We found that the targeted disruption of the two isoforms of the *ATP synthase subunit 6* gene, *atp6‐1* and *atp6‐2*, using a TALEN‐based editor was efficient, and the mitochondrial genomes were recovered in a homoplasmic state (Arimura et al., 2020). This homoplasmy means that all copies of the mitochondrial genomes (10^1^–10^2^ copies/cell) have the same genotype. However, the targeted mutagenesis on NUMTs is challenging (Zhong, 2025). This observation led us to hypothesize that methylation inhibits mutagenesis efficiency in the nucleus. In this study, we fixed the target sequence at the *FWA* gene locus and investigated the effect of CG methylation on mutagenesis efficiency, specifically comparing CRISPR/Cas9 and TALEN editors. Our results showed that 5mC negatively affected mutagenesis efficiencies for both editors in both the promoter and gene body of *FWA*. Overall, TALEN is robust and performed comparably to CRISPR/Cas9 at the tested sites. We then developed ngTALEN to target 5mC and achieved improved editing in plants. By comparing the effectiveness of three editors, CRISPR/Cas9, TALEN, and ngTALEN, in editing overlapping windows at the *FWA* locus with different 5mC statuses in both Col‐0 and *fwa* epimutant background, we reported efficient editing by ngTALEN on methylated DNA and showed that the editing was heritable.

## RESULTS

### Identification of targeting sites on the *FWA* gene locus with varied numbers of 5mC modifications

**Figure 1:**
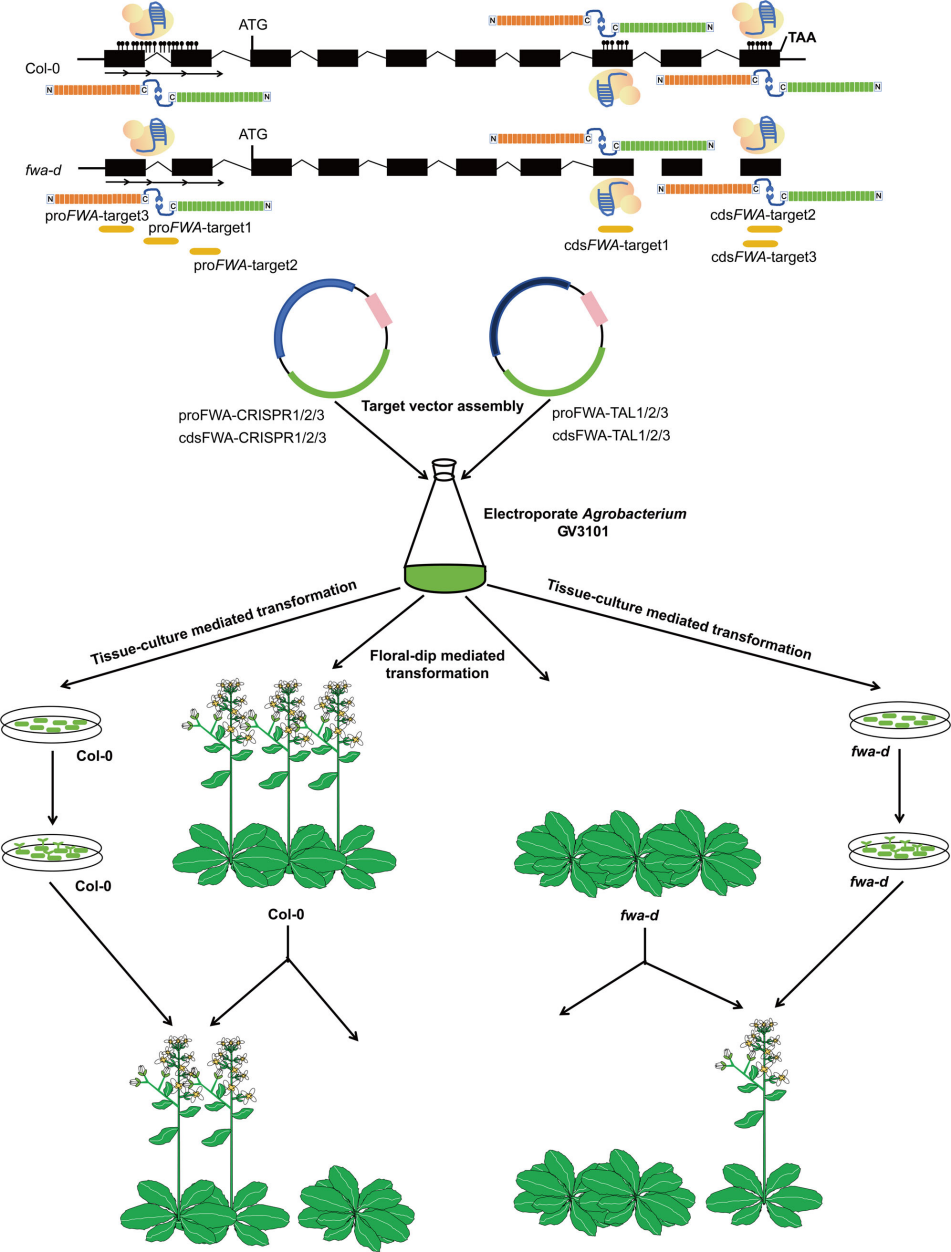
Editing targets on *FWA* and the schematic design of genetic transformation. Schematic illustration of targets for CRISPR/Cas9 and TALEN on the *FWA* promoter and CDS regions in the Col‐0 and *fwa‐d* epimutant backgrounds. The genomic structure of *FWA* is depicted with boxes representing exons and bent lines indicating introns. Two exons located within the two direct repeats (indicated by two sets of arrows) in the promoter of *FWA* are shown below. Cartoons of CRISPR/Cas9 and TALEN are placed in opposite directions in parallel to showcase their overlapping target windows. Three targets in the promoter and another three in the CDS regions corresponding to exon 6 and exon 8 are depicted as green bars bellow. Introduction of genome editors into the nucleus was mediated by floral‐dipping and tissue culture methods. Editing was detected by amplicon Sanger sequencing, and the homozygous edits were screened for the late‐flowering phenotype.

To exclude the possible impact of sequence composition of target sites on editing efficiency, six sites with differential 5mCs compositions were selected in the two tandem‐repeat promoter regions and the coding DNA sequence (CDS) regions corresponding to exon 6 and exon 8 on the *FWA* locus (Figure 1). To clearly analyze the influence of 5mC on editing efficiency and identify potential solutions for dealing with 5mC, CRISPR/Cas9, TALEN, and ngTALEN were targeted to each of the six sites with overlapping editing windows in two backgrounds (Col‐0 and *fwa‐d*), generating a total of almost 3 × 6 × 2 = 36 transformed materials. The editor‐expression vectors were introduced separately into the nuclei of wild‐type *A. thaliana* Col‐0 and the epimutant *fwa‐d*. Introduction of genome editors into the nucleus was mediated by the floral‐dipping method, which is the most common transformation method used in *Arabidopsis*. In Col‐0 plants, *FWA* is expressed in the diploid central cell prior to fertilization and in the endosperm in an imprinted manner (Kinoshita et al., 2004). To mitigate the possible influence on genome editing by the transient expression of *FWA* in gametophyte cells in Col‐0 when floral‐dipping transformation was conducted, tissue culture‐mediated editor transformation was also performed at the same time. Editing in the promoter might change the expression of *FWA*, while editing in the C‐terminal region of the protein might affect interaction with FLOWERING LOCUS T (FT), thereby causing altered flowering phenotypes.

Because TALEN is more flexible in positioning than CRISPR/Cas9, the overlapping editing targets were determined by CRISPR/Cas9. CRISPOR software (Concordet & Haeussler, 2018) (https://crispor.gi.ucsc.edu/) was utilized to screen sgRNAs targeting the two tandem‐repeat regions of the *FWA* promoter, resulting in a total of 30 sgRNA candidates. A pipeline was developed to further filter these sgRNAs based on parameters, such as high efficiency, absence of potential off‐targets in the genome, and coverage of at least one methylated cytosine. Ultimately, three sgRNAs within the promoter were selected for editing analysis. Similar approaches were applied to screen sgRNAs targeting methylated CDS regions within the *FWA* gene body, also yielding three sgRNAs (Figure S1A,B). By comparing the number of 5mCs in the overlapping editing window and the flanking TALEN binding sequences, it was found that the three targets at the promoter were located in hypermethylated regions with approximately 21 5mCs, whereas the other three targets in the gene body of the *FWA* locus were in less methylated CDS regions with only seven 5mCs. The editing efficiency, binding position, methylation status, and possible off‐targets were predicted by CRISPOR for all the six sgRNAs. To evaluate editing scores, *Chelatase 12* (*CHLI2*, AT5G45930), located in euchromatin known for its high editing efficiency with CRISPR/Cas9 (Weiss et al., 2022), was introduced as a positive control and also used in subsequent experiments. The predicted editing efficiency for *CHLI2* scored 65, which was comparable to most of the sgRNAs, which scored 60, 61, 65, 67, and 69, with the exception of proFWA‐CRISPR1, which had a score of 46. Overall, the six selected sgRNAs were predicted to be effective and covered varying numbers of heavily methylated cytosines (Figure S1C). Given that the editing window has been fixed by CRISPR/Cas9, the TALEN arms flanking the editing window were then designed based on several criteria. These included ensuring that the recognition of TALEN pairs to nucleobases started from a base T and that the minimum recognition sequence was 15 bp in length to ensure specifically binding to the target sequence. Furthermore, the TALEN Targeter “old version with design guidelines” (https://tale‐nt.cac.cornell.edu/node/add/talen‐old) (Doyle et al., 2012), was utilized to evaluate the pairs of TALEN arms flanking the sgRNA binding sites. The scoring system is based on summing the negative logs of the appropriate RVD‐nucleotide association probabilities (Doyle et al., 2012). Consequently, better alignments result in lower scores. The tool returns target scoring below a threshold cutoff, defined as a ratio of observed score to the best possible score, of 3.0 or less. Based on these criteria, the TALENs were predicted to be suboptimal, as most of the arms scored above 3.0.

### TALEN and CRISPR/Cas9 editing was inhibited by CG methylation at less methylated CDS regions of *FWA* in tissue culture regenerated transformants

Next, we evaluated the mutagenesis efficiency of the three editors, CRISPR/Cas9, TALEN, and ngTALEN, for each of the six targeting sites in two genetic backgrounds, Col‐0 and epinutant *fwa*. Ti‐plasmids containing a CRISPR/Cas9 (TALEN or ngTALEN pairs) expression cassette, a seed‐specific expression proOle‐GFP reporter, and the *NPTII* selection marker gene were constructed. A representative arm of DNA recognition motifs for the left arm of one of the TALEN constructs, named cdsFWA‐TALEN1 (cdsFWA‐TALEN1L; “L” referred to as “left”), is shown in the middle of Figure S2A, and the complete list of target sequences of the editors is also provided in Figure S2B. The constructs were designated according to the following nomenclature: (promoter or cds)FWA‐(CRISPR, TALEN, or ngTALEN)(target number) in (Col‐0 or *fwa‐d* background). A BLAST search did not reveal any off‐target sites across the entire genome.

**Figure 2:**
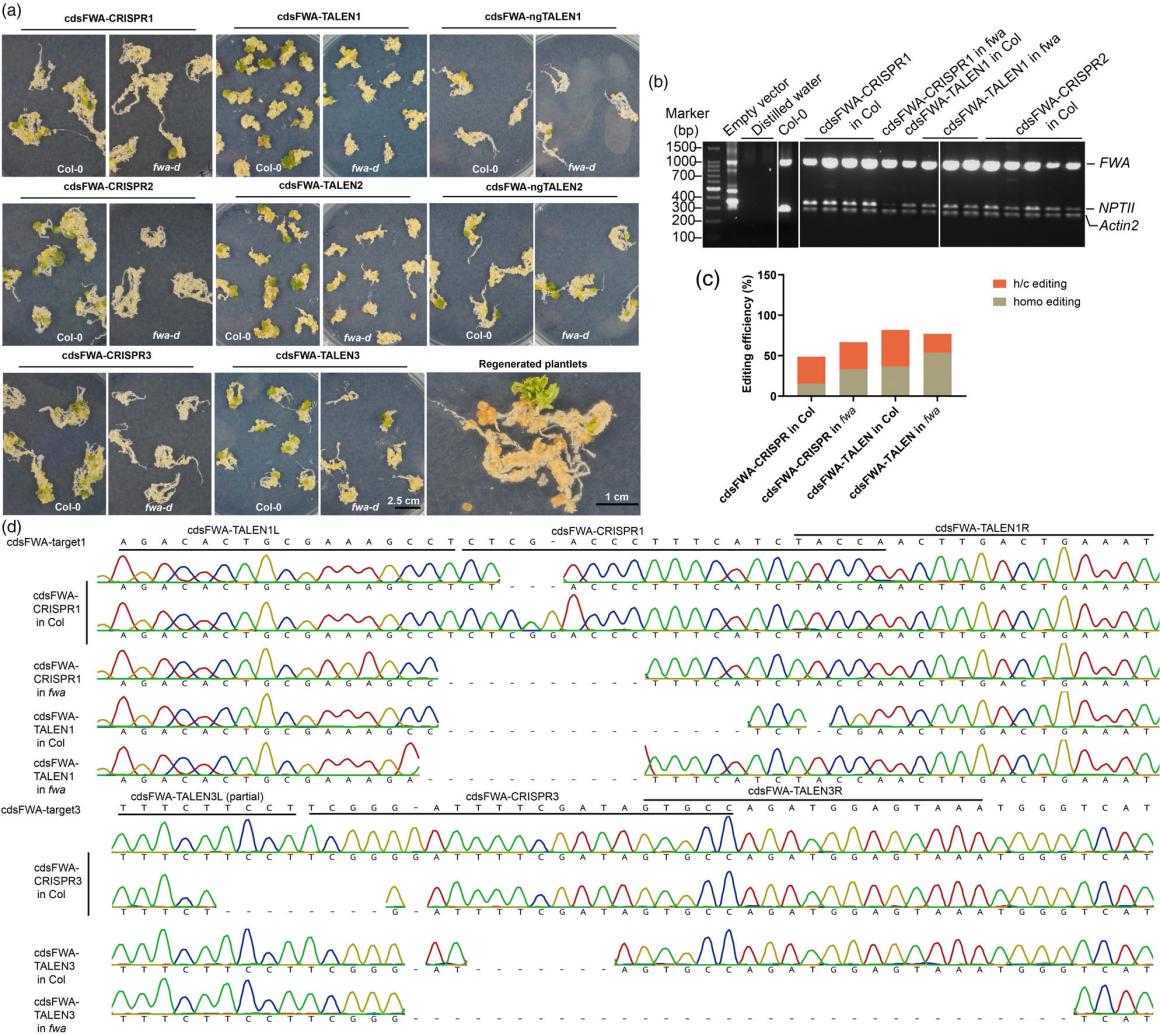
Comparison of editing frequency of editors targeting the *FWA* locus in regenerated plantlets. (a) Embryogenic calli induction and plant regeneration for editors targeting the CDS region of *FWA*. (b) Multiplex PCR (simultaneous amplification of multiple targets in a single reaction mixture) was performed to co‐amplify *FWA*, *NPTII*, and *Actin2*. Amplification of *NPTII* indicates the presence of genome editors in the nuclei, while *Actin2* served as a loading control. The empty vector contains *NPTII*, and its amplification serves as a positive control, with distilled water serving as the negative control. (c) Summary of editing frequencies on target windows in regenerated T_0_ plantlets with Col‐0 or *fwa* epimutant backgrounds. Abbreviations: homo, homozygous editing; h/c, heterozygous/chimera editing. (d) Representative Sanger sequencing results showing different types of homozygous mutations induced in the target window by genome editors in regenerated plants. The wild‐type sequence is provided at the top. For CRISPR/Cas9, mutations included 1‐bp insertions or 2‐ to 9‐bp deletions. For TALEN and ngTALEN, mutants exhibited 7‐ to 31‐bp deletions.

Seventeen vectors were constructed and introduced into wild‐type Col‐0 and epimutant *fwa* through tissue culture‐mediated transformation of root explants. Three months later, the target regions in the regenerated plants were amplified and Sanger sequenced. Although we failed to regenerate plants from the embryogenic calli for almost all of the editor transformants targeting *FWA* promoter regions (Figure S3A,B), we did obtain regenerated calli and limited numbers of embryogenic calli. In contrast, a bunch of regenerated plantlets was obtained from embryogenic calli for editor transformants targeting the CDS regions of *FWA* (Figure 2a). This indicates that editing in the promoter region might interfere with the methylation or expression of *FWA*, causing activation of this gene and thus inhibiting regeneration (Dai et al., 2021). Multiplex PCR (amplifying multiple targets simultaneously in a single reaction mixture) was conducted to co‐amplify fragments for *FWA*, *NPTII*, and *Actin2* in the regenerated plantlets. The amplification of *NPTII* served as a double‐check for GFP‐positive seeds, indicating the presence of genome editors in the plantlets (Figure 2b). The genomic PCR bands covering the coding region from exon 6 to exon 8 appeared in primary regenerated plantlets, suggesting that short indels rather than large insertions or deletions might have occurred.

One leaf from each of the regenerated plantlets was detached and subjected to DNA extraction, PCR amplification, and Sanger sequencing. Only three plantlets were regenerated for the editor transformants targeting the promoter of *FWA*, whereas 66 plantlets were regenerated from the editor transformants targeting CDS regions of *FWA*. Sanger sequencing of the amplicons revealed that the frequency of homozygous editing (homo) within the target window was comparable in TALEN transformants compared with CRISPR/Cas9 transformants, demonstrating the robustness of TALEN editors (Figure 2c). Subsequently, we analyzed whether the editing frequencies were correlated with 5mC modification and found that the frequency of homozygous editing in the *fwa* epimutant background was substantially higher than in the Col‐0 background. A pairwise analysis of the editing in the CDS regions of *FWA* in the regenerated plantlets showed homozygous editing frequencies of 15.38% for cdsFWA‐CRISPR in Col‐0 transformants, 33.33% for cdsFWA‐CRISPR in *fwa* transformants, 36.36% for cdsFWA‐TALEN in Col‐0 transformants, and 53.85% for cdsFWA‐TALEN in *fwa* transformants, respectively (Figure S3C,D). These results suggest that 5mC modification in the gene body of *FWA* inhibits editing efficiencies. Although most of the transformants in the first generation exhibited heterozygous or chimeric mutations (h/c), homozygous edits could be obtained in subsequent generations. Therefore, we included h/c editing in our calculation of total editing efficiency. Representative Sanger sequencing for homozygous editing was presented (Figure 2d). CRISPR/Cas9‐induced mutations encompassed 1 bp insertion and 2–9 bp deletions, whereas TALEN‐induced mutations comprised 7–31 bp deletions in the homozygously edited regenerants.

### TALEN and CRISPR/Cas9 editing was inhibited by CG methylation at less methylated CDS regions of *FWA* in floral‐dipping transformants

**Figure 3:**
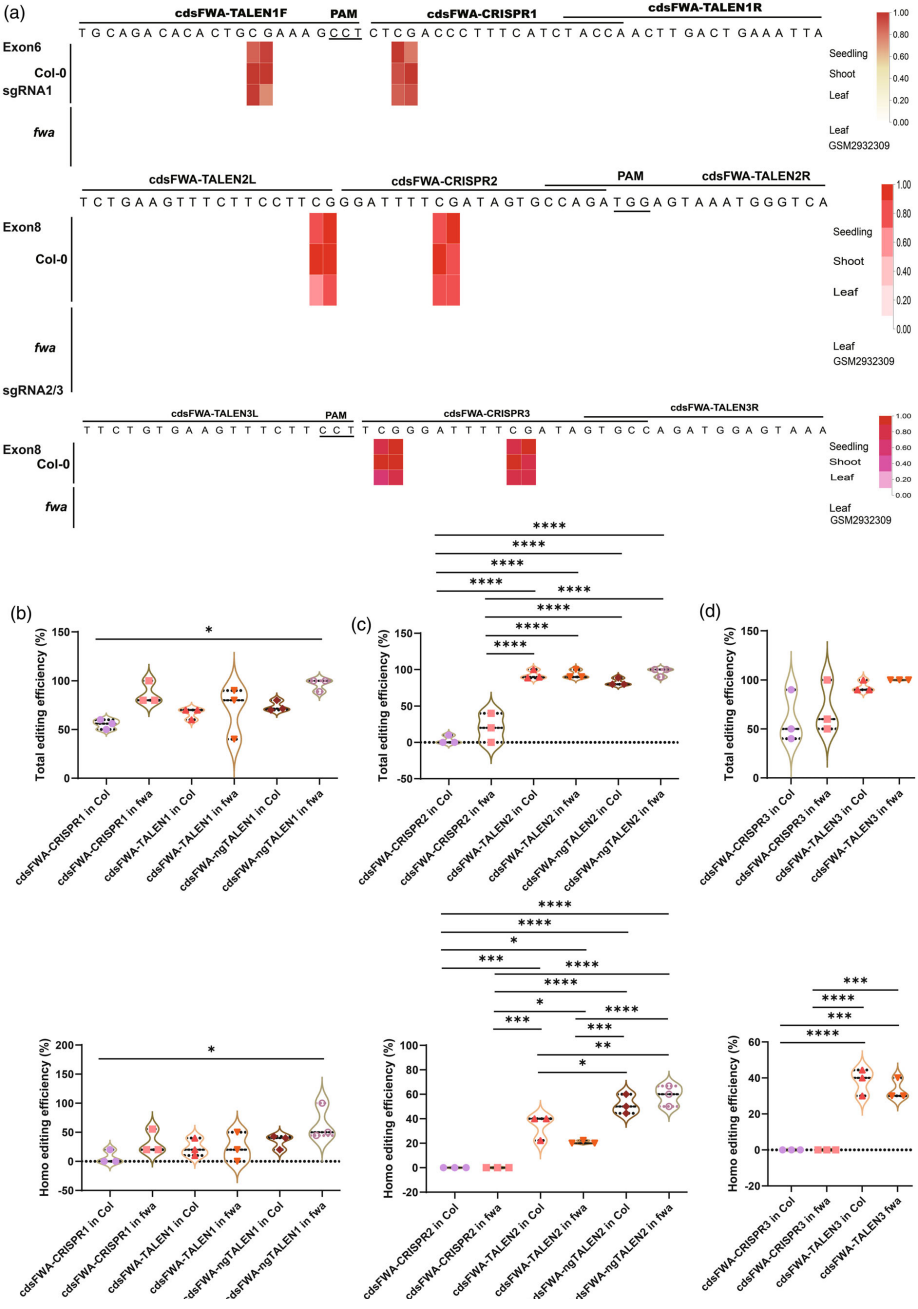
Comparison of the editing efficiency of editors on less methylated CDS regions of *FWA*. (a) DNA methylation levels at the three target sites within exon 6 and exon 8 CDS regions of *FWA* in Col‐0 and *fwa* epimutant. Multiple whole genome bisulfite sequencing (WGBS) data for seedling, shoot, and leaf tissues at the targeting loci in Col‐0, and for leaf tissue in *fwa* epimutant (GEO accession no. GSM2932309), were obtained from the AraENCODE database V1.1 and re‐visualized as a heatmap. Methylation levels are normalized to a color scale ranging from 0 to 1, with 1 indicating the highest level (darkest color) and 0 indicating the lowest level (white). (b–d) Violin plot displaying the total editing and homozygous editing frequencies at the cdsFWA‐target1 (b), cdsFWA‐target2 (c), and cdsFWA‐target3 (d) sites in T_1_ plants with Col‐0 or *fwa* epimutant backgrounds. *P < 0.05, **P < 0.01, ***P < 0.001, ****P < 0.0001.

In general, there were far fewer 5mCs in the gene body of *FWA* than in the promoter region. Only the corresponding CDS regions of exon 6 and exon 8 were methylated, with five CG methylations in exon 6 and two CG methylations in exon 8, respectively (Figure S4A). We compared methylation levels of targeting sites at the exon 6 and exon 8 CDS regions of *FWA* using the AraENCODE database (Wang et al., 2023) in Col‐0 and *fwa* epimutant. Whole genome bisulfite sequencing (WGBS) data from multiple Col‐0 samples (seedling, shoot, and leaf tissues) and one *fwa* epimutant sample (leaf tissue, GSM2932309) were publicly available and visualized on the AraENCODE database. We focused our analysis on the target CDS regions of *FWA* and drew a heatmap to re‐visualize the methylation levels. In Col‐0, there was only one 5mC at each of the cdsFWA‐CRISPR1 and cdsFWA‐CRISPR2 targeting sites, whereas the cdsFWA‐CRISPR3 targeting site had two hypermethylated 5mCs (Figure 3a). Similarly, there was only one 5mC on each of the left arms of cdsFWA‐TALEN1 and cdsFWA‐TALEN2, whereas cdsFWA‐TALEN3 binding sites did not contain any 5mC. This enabled us to compare editing efficiency at targets with varied levels of 5mC modifications. It is evident that the 5mCs across the target regions were predominantly CG methylation. In contrast, we observed complete demethylation at all corresponding targets in the *fwa* epimutant.

To confirm that 5mC, particularly CG methylation, at gene body inhibits editing, we transformed Ti‐plasmids expressing editors into *A. thaliana via* floral‐dipping method. Across all three target regions, the editing frequency was significantly higher for editors in the *fwa* background compared with Col‐0 background, confirming that CG methylation inhibits genome editing in the gene body of *FWA* (Figure 3b–d). In these less methylated CDS regions, TALEN editing frequency was almost equivalent to that of CRISPR/Cas9 in editor transformants with a Col‐0 background, and ngTALEN further improved editing efficiency (Figure S4B). The restoration of the *fwa* late‐flowering phenotype by editor transformants in the *fwa* background was observable on 1/2 MS plates after 3 weeks (Figure S4C). This reversal of the phenotype may be caused by the editing of the C‐terminal domain of the FWA protein. Deletion in the C‐terminal domain may inhibit physical interaction with FT (Ikeda et al., 2007), thereby derepressing FT function and reversal of the late‐flowering phenotype in the *fwa* epimutant background. The pattern was consistent and observable in both total editing and homozygous editing events. For instance, the total editing frequency for cdsFWA‐CRISPR1 in Col transformants was 54.17%, for cdsFWA‐CRISPR1 in *fwa* transformants was 86.21%, for cdsFWA‐TALEN1 in Col transformants was 66.67%, for cdsFWA‐TALEN1 in *fwa* transformants was 70%, for cdsFWA‐ngTALEN1 in Col transformants was 74.07%, and for cdsFWA‐ngTALEN1 in *fwa* transformants was 92.86% (Figure S4D). However, we observed suboptimal improvement for ngTALEN compared with TALEN, which may be due to the lower level of 5mCs in these CDS regions of the *FWA* locus. When analyzing all three targets combined, the total editing frequency for cdsFWA‐CRISPR in Col transformants was 38.10%, for cdsFWA‐CRISPR in *fwa* transformants was 58.43%, for cdsFWA‐TALEN in Col transformants was 84.10%, for cdsFWA‐TALEN in *fwa* transformants was 87.50%, for cdsFWA‐ngTALEN in Col transformants was 78.57%, and for cdsFWA‐ngTALEN in *fwa* transformants was 95.35% (Figure S4E).

### Inheritance and stability of the *FWA* CDS edited mutants

**Figure 4:**
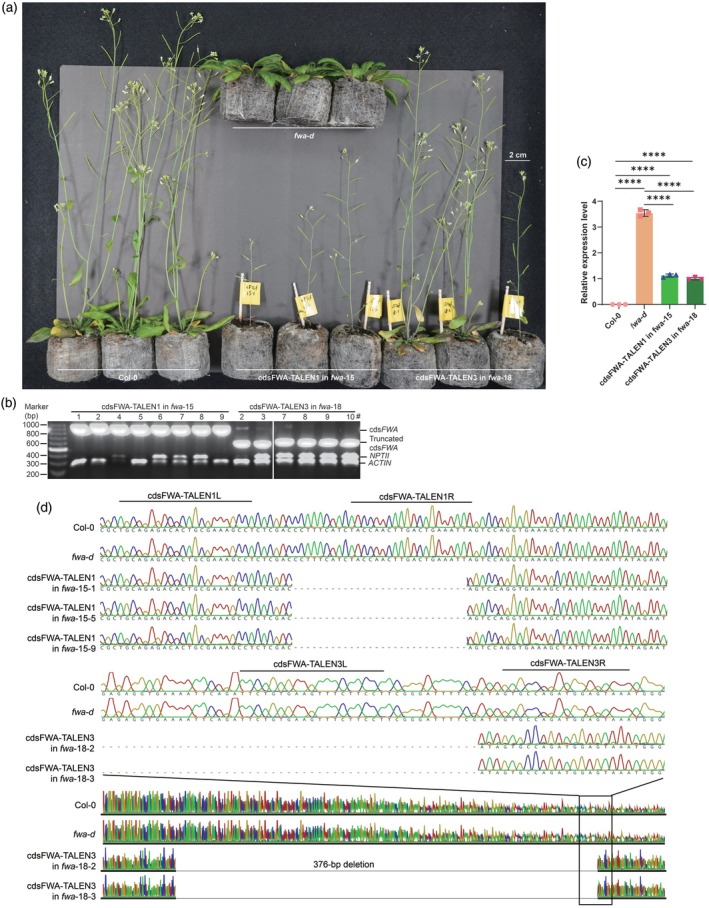
TALEN and ngTALEN editing of the *FWA* gene body results in the heritable reversal of the *fwa* late‐flowering phenotype. (a) A representative flowering phenotype of 5‐week‐old T_2_ edited mutants grown under long‐day conditions is shown. (b) Genotyping results for TALEN and ngTALEN in T_2_ transformants are presented with multiplex genomic PCR results for selected edited lines. Some edited plants have segregated away the transgene without *NPTII* expression. (c) A bar graph depicts the *FWA* expression in edited T_2_ plants, where each dot represents data from one sample per replicate. A one‐way ANOVA test indicated that *FWA* expression was significantly upregulated in the *fwa‐d* mutant but suppressed in Col‐0, cdsFWA‐TALEN1 in *fwa*‐15, and cdsFWA‐TALEN3 in *fwa*‐18 leaves from 2‐week‐old plants. (d) Representative Sanger sequencing results demonstrate the stable and heritable mutations induced by the genome editors in T_2_ edited plants. The wild‐type sequences of Col‐0 and *fwa* are provided at the top. Lines from cdsFWA‐TALEN1 in *fwa*‐15 exhibit a 28‐bp deletion, while other offspring derived from selfing of cdsFWA‐TALEN3 in *fwa*‐18 show a 376‐bp deletion in the C‐terminus of the *FWA* CDS region. ****P < 0.0001.

We previously found that mutations in the nucleus created by TALE‐based editors were stably fixed and inherited throughout plant growth and development (Hosoda et al., 2024). To examine the inheritance of the targeted mutations and the introduced vectors for genome editing, we sowed seeds from T_1_ individuals that had homozygous edits with observable phenotype changes. Targeted disruption of the gene body of *fwa* caused a partial reversal of the late‐flowering phenotype in T_1_ edited plants. We then studied the stability of the editing events over generations by analyzing the flowering time. Representative T_2_ plants derived from the T_1_ generation named cdsFWA‐TALEN1 in *fwa*‐15 and cdsFWA‐TALEN3 in *fwa*‐18, which carried a 28‐bp deletion and a 376‐bp deletion in the target window, respectively, partially reversed the late‐flowering phenotype of *fwa* (Figure 4a). Multiplex PCRs revealed that some of these T_2_ plants, cdsFWA‐TALEN1 in *fwa*‐15‐1/2/5/9 and cdsFWA‐TALEN3 in *fwa*‐18‐2, lacked the *NPTII* transgene in the nucleus (Figure 4b). This indicates that no additional editing should have occurred in these plants.

To test whether the restoration of the late‐flowering phenotype observed was due to *FWA* downregulation, we performed qRT‐PCR analysis for verification. Expression of *FWA* in Col‐0 was almost undetectable, but it was highly upregulated in the *fwa* epimutant. The expression of *FWA* was significantly downregulated in the transgene‐free T_2_ plants described above (Figure 4c). This result is interesting because the editing occurred in the gene body of *FWA*, whose modification was not expected to alter its expression. Nevertheless, downregulation of *FWA* expression contributes to the partial reversal of the *fwa* late‐flowering phenotype in the edited plants. They inherited deletion patterns similar to those of the parental lines (Figure 4d). Together, these results suggest that the deletion in the *FWA* gene body may indirectly compromise expression of *FWA* and restore the *fwa* late‐flowering phenotype. The newly generated mutants can be stably inherited, even in the absence of the editors in the nuclei.

### ngTALEN improved editing at hypermethylated promoter regions of *FWA*

**Figure 5:**
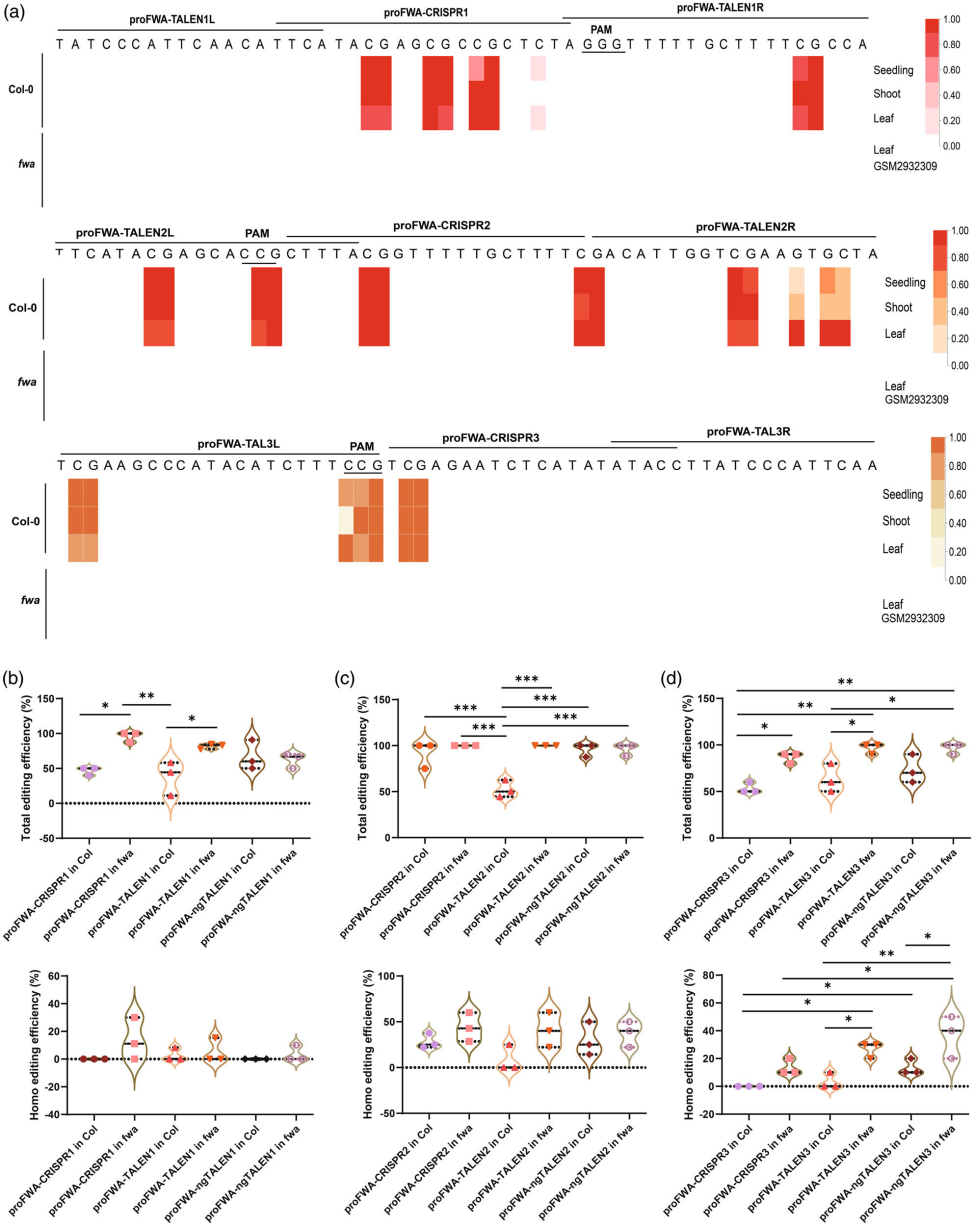
Comparison of the editing efficiency of editors on highly methylated promoter regions of *FWA*. (a) DNA methylation levels over the target sites in the two direct repeat regions of the *FWA* promoter in Col‐0 or *fwa* epimutant. Multiple WGBS data for seedling, shoot, and leaf tissues at the targeting locus in Col‐0, and for leaf tissue in *fwa* epimutant (GEO accession no. GSM2932309), were collected from AraENCODE database V1.1 and re‐visualized as a heatmap. Methylation levels are normalized to a color scale of 0–1, with 1 indicating the highest level (darkest color) and 0 indicating the lowest level (white). (b–d) Violin plot showing the total editing and homozygous editing frequencies at the proFWA‐target1 (b), proFWA‐target2 (c), and proFWA‐target3 (d) sites in T_1_ plants with Col‐0 or *fwa* epimutant background. For the violin plots, each data point represents the editing frequency for 10 plants, representing a repeat for a specific editor. Abbreviations: homo, homozygous editing; h/c, heterozygous/chimera editing. The middle dashed line represents the mean, and the other dashed lines represent the interquartile ranges. *P*‐values were calculated using one‐way ANOVA multiple comparison tests. *P < 0.05, **P < 0.01, ***P < 0.001.

Methylation in the two direct repeat regions of the *FWA* promoter is responsible for suppressing the expression of this gene and maintaining normal flowering time in Col‐0 plants (Kinoshita et al., 2007). There were approximately 21 CG methylation sites in these two direct repeats, with most of them being heavily methylated (Figure S5A). We also profiled the methylation levels of targeting sites within the two direct repeat regions of the *FWA* promoter using the AraENCODE database in both Col‐0 and *fwa* epimutant. In Col‐0, we found three closely hypermethylated 5mCs and one less methylated 5mC on proFWA‐CRISPR1, one hypermethylated 5mC on proFWA‐CRISPR2 located 6 bp away from the methylated PAM site, and one hypermethylated 5mC on proFWA‐CRISPR3 located 2 bp away from the methylated PAM site (Figure 5a). Similarly, there was only one 5mC on the right arm of proFWA‐TALEN1, two and four 5mCs on the left and right binding sites of proFWA‐TALEN2, respectively, and three 5mCs on the left arm of the proFWA‐TALEN3 binding site. In contrast, complete demethylation was observed at all three corresponding targets in the *fwa* epimutant.

Consistent with editing in the gene body of *FWA*, editing frequencies for all three target windows in the promoter were significantly higher in editor transformants with an *fwa* background compared with those with a Col‐0 background. This confirms that CG methylation inhibits genome editing not only within the gene body but also in the promoter of *FWA* (Figure 5b–d). In these hypermethylated promoter regions, the TALEN editing was equivalent to those of CRISPR/Cas9, whereas ngTALEN further improved the editing efficiency of TALEN and performed best among the three editors in the Col‐0 background (Figure S5B). An exemplary reversal of the *fwa* late‐flowering phenotype by proFWA‐TALEN1 in *fwa*‐12 was shown in T_1_ generation (Figure S5C,D). This reversal of the phenotype may have been caused by editing of the promoter, leading to downregulation of *FWA* and suppression of late‐flowering in the *fwa* epimutant background. The pattern was consistent and observable in both total editing and homozygous editing events. For example, the homozygous editing frequency for proFWA‐CRISPR3 in Col transformants was 0.00%, whereas in *fwa* transformants, it was 13.33%. Similarly, for proFWA‐TALEN3 in Col transformants, it was 3.33%, but in *fwa* transformants, it was 26.67%. For proFWA‐ngTALEN3 in Col transformants, it was 13.33%, and in *fwa* transformants, it was 36.67% (Figure S5E). Furthermore, we observed improvements of editing efficiency with ngTALEN compared with TALEN, potentially due to the abundance of 5mCs in these promoter regions of the *FWA* locus. However, ngTALEN in the *fwa* epimutant background exhibited reduced efficacy due to the mismatches between ngTALEN and non‐methylated cytosines. This trend was also evident in the combined analysis of all three targets. For example, the total editing frequency for proFWA‐CRISPR in Col transformants was 60.92%, whereas in *fwa* transformants, it was 93.51%. For proFWA‐TALEN in Col transformants, it was 50.59%, but in *fwa* transformants, it was 93.11%. For proFWA‐ngTALEN in Col transformants, it was 76.83%, and in *fwa* transformants, it was 82.93% (Figure S5F).

### Inheritance and stability of the *FWA* promoter edited mutants

**Figure 6:**
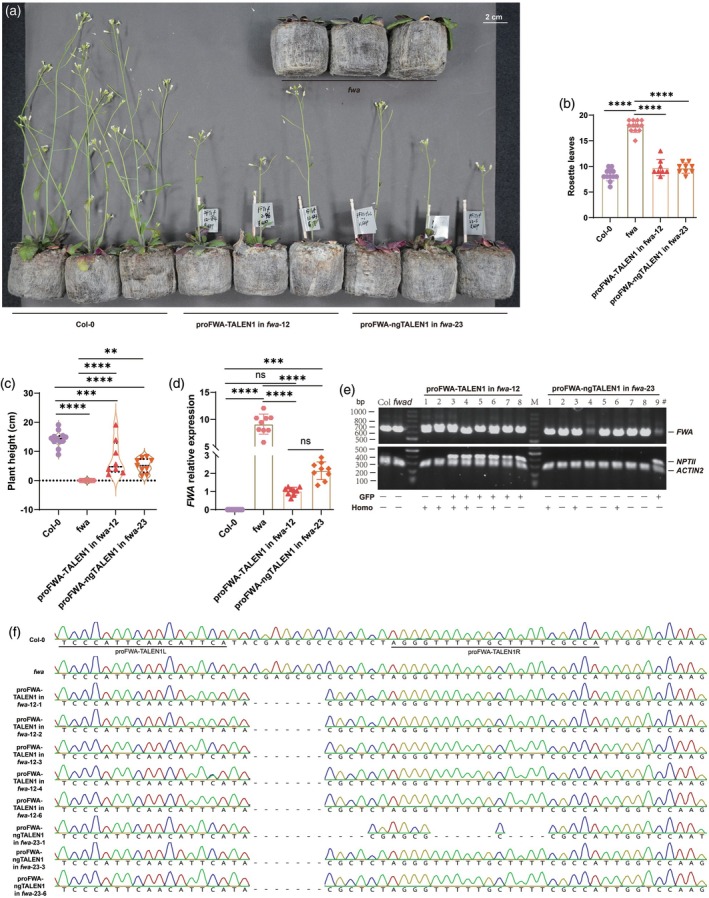
TALEN and ngTALEN editing of the *FWA* promoter results in heritable restoration of the *fwa* late‐flowering phenotype. (a) Representative flowering phenotype of 5‐week‐old T_2_ edited *FWA* mutants grown under long‐day conditions. (b) Flowering time was examined by counting the number of rosette leaves. Data for individual plants are depicted as colored dots. (c) Plant height, as an additional demonstration of the restoration of the *fwa* phenotype through targeted editing. Data for individual plants are depicted as colored dots. (d) Bar graph showing *FWA* expression in edited T_2_ plants. Each sample includes three biological replicates and three technical replicates. Each dot represents data for one sample per replicate. One‐way ANOVA test suggested that *FWA* expression was highly upregulated in the *fwa‐d* mutant but suppressed in Col‐0, proFWA‐TALEN1 in *fwa*‐12, and proFWA‐ngTALEN1 in *fwa*‐23, in leaves of 2‐week‐old edited plants. (e) Representative genotyping of TALEN and ngTALEN in T_2_ transformants. Multiplex genomic PCR results for representative edited lines are shown. Some of the edited plants have segregated away the transgene without *GFP* and *NPTII* expression. (f) Representative Sanger sequencing results demonstrating the stable and heritable mutations induced by the genome editors in T_2_ edited plants. The wild‐type sequences of Col‐0 and *fwa* are provided at the top. Apart from proFWA‐ngTALEN1 in *fwa*‐23‐1, which had a 21‐bp deletion, the other offspring derived from selfing of proFWA‐TALEN1 in *fwa*‐12 and proFWA‐ngTALEN1 in *fwa*‐23 were mutated with the same 7‐bp deletion. **P < 0.01, ***P < 0.001, ****P < 0.0001.

The *FWA* promoter is normally hypermethylated in Col‐0, causing silencing of *FWA* and early flowering. Demethylation of the promoter in *fwa* epiallele triggers ectopic expression of *FWA* and late flowering (Gallego‐Bartolomé et al., 2018). Targeted disruption of the promoter of *fwa* caused a partial restoration of the normal flowering phenotype in T_1_ edited plants. We then studied the stability of the editing events over generations by analyzing the flowering time. Representative T_2_ plants carrying proFWA‐TALEN1 in *fwa*‐12 and proFWA‐ngTALEN1 in *fwa*‐23, both with 7‐bp deletions in the target window, partially reversed the late‐flowering phenotype of *fwa* (Figure 6a–c; Figure S6A,B).

To test whether the reversal of the late‐flowering phenotype observed was due to *FWA* downregulation, we performed qRT‐PCR analysis for verification. Expression of *FWA* in Col‐0 was almost undetectable, but it was highly upregulated in the *fwa* epimutant. The expression of *FWA* in the two TALEN and ngTALEN edited lines, proFWA‐TALEN1 in *fwa*‐12 and proFWA‐ngTALEN1 in *fwa*‐23, was significantly downregulated (Figure 6d). This result is in good agreement with the partial reversal of the *fwa* late‐flowering phenotype in the edited plants. To confirm the presence of editors, selfed T_2_ plants from representative T_1_ lines with deletions in proFWA‐TALEN1 in *fwa*‐12 and proFWA‐ngTALEN1 in *fwa*‐23 were analyzed by multiplex PCRs (Figure 6e). Siblings, including proFWA‐TALEN1 in *fwa*‐12‐1/2 and proFWA‐ngTALEN1 in *fwa*‐23‐1/3/6, were homozygous lines without *GFP* and *NPTII* expression. They inherited deletion patterns similar to those of the parental lines (Figure 6f). Furthermore, T_3_ null‐segregant progenies without the editor in the nuclei were obtained, showing a wild‐type‐like phenotype (Figure S6C,D). Several other edited lines showing reversal of the *fwa* late‐flowering were presented as well (Figure S6E). The reversal of the mutant phenotype might be due to the disruption of the key *cis*‐elements in the promoter (i.e., TATA‐box) or interference of interaction with other proteins (i.e., FT) in the C‐terminus of FWA protein (Figure S6F). It is unlikely that promoter editing increased methylation levels, thereby suppressing *FWA* expression and causing the reversal of the late‐flowering phenotype. Indeed, bisulfite sequencing showed that the overall methylation status of the two tandem repeat promoter region of *FWA* in the edited plants has not changed. Additionally, detailed phenotyping, expression of *FWA*, and genotyping using multi‐PCR were further performed on the T_3_ edited plants, showing consistency with the T_2_ generation (Figure S7). Together, these results suggest that the deletion in the *FWA* promoter may disrupt its regulatory activity, resulting in compromised expression of *FWA* and reversal of the *fwa* late‐flowering phenotype. The newly generated mutants can be stably inherited, even in the absence of the editors in the nuclei.

### ngTALEN was suboptimal in editing sites with complex chromatin features

When combining sequencing data for approximately 1000 samples for all six targeting sites of *FWA*, regardless of whether CG methylation was high or low, a consistent editing pattern emerged for the editors. Specifically, CG methylation inhibited the editing efficiency for both TALEN and CRISPR/Cas9. TALEN editing efficiency is equivalent to CRISPR/Cas9, and ngTALEN exhibits improved editing than TALEN on CG‐methylated sites in the Col‐0 background (Figure S8A). This pattern of editing efficiency was observed in both homozygous editing and total editing frequencies (Figure S8B). For instance, the total editing frequencies were as follows: 49.71% for FWA‐CRISPR in Col transformants, 74.70% for FWA‐CRISPR in *fwa* transformants, 67.63% for FWA‐TALEN in Col transformants, 90.29% for FWA‐TALEN in *fwa* transformants, 77.54% for FWA‐ngTALEN in Col transformants, and 87.20% for FWA‐ngTALEN in *fwa* transformants. Notably, all of the edited plants that exhibited a reversal of the late‐flowering phenotype were generated by TALEN (two lines) or ngTALEN editors (eight lines), but not by CRISPR/Cas9. This may be due to the relatively larger fragment deletions caused by TALEN/ngTALEN, which disrupted the key *cis*‐elements in the promoter of *FWA*, leading to the downregulation of *FWA* expression and the reversal of the late‐flowering phenotype.

**Figure 7:**
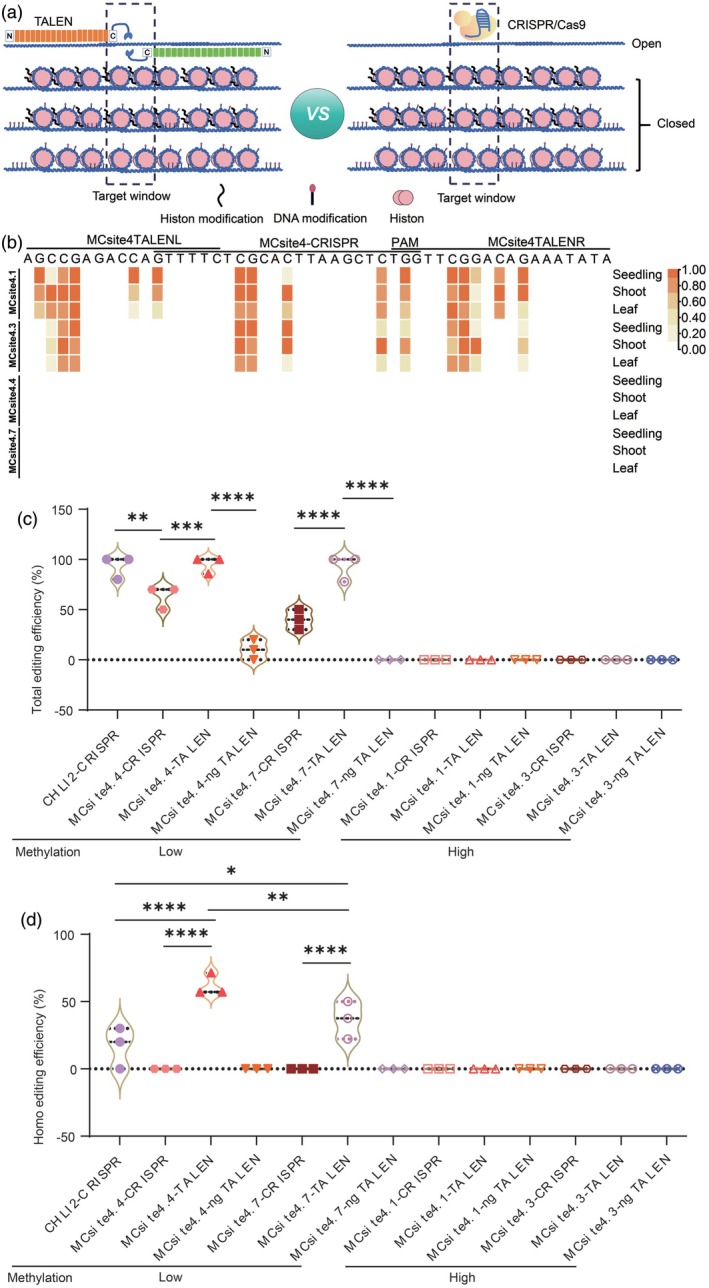
Comparison of editing efficiency of editors on intergenic regions with multiple chromatin features. (a) Schematic representation of genome editors targeting multiple intergenic regions with DNA methylation and various chromatin features. (b) DNA methylation levels across the target sites of MCsite4 in Col‐0. Multiple WGBS data for seedling, shoot, and leaf tissues at the targeting locus in Col‐0 were sourced from the AraENCODE database V1.1 and re‐visualized in a heatmap. Methylation levels are normalized to a color scale of 0–1, with 1 indicating the highest level (darkest color) and 0 indicating the lowest level (white). (c, d) Violin plot showing the total (c) and homozygous (d) editing frequencies for the four targets on MCsites4 in T_1_ editor transformants. For the violin plots, each data point represents the editing frequency for a set of 10 plants, representing a repeat for a specific editor. h/c, heterozygous/chimera editing; homo, homozygous editing. The middle dashed line signifies the means. The other dashed lines represent the interquartile ranges. *P*‐values were calculated by one‐way ANOVA multiple comparison tests. *P < 0.05, **P < 0.01, ***P < 0.001; ****P < 0.0001.

Although CG methylation is the major cause of gene silencing, non‐CG methylation can also have significant consequences (Dai et al., 2021). To test the ability of ngTALEN to target the same sequence across the genome in the context of distinct DNA methylation patterns and diverse chromatin environments, such as 5mC (CG, CHG, and CHH), histone modifications (e.g., H3K9me2), and chromatin accessibility, we directed the editors to multicopy CRISPR site 4 (MCsite4) (Weiss et al., 2022), which is predominantly located in intergenic regions (Figure 7a). We analyzed methylation levels of targeting sites at MCsite4 using the AraENCODE database in Col‐0 and re‐visualized the data using a heatmap. Overall, there were two heavily methylated sites (MCsite4.1: heterochromatin, no detectable RNA, not accessible; and MCsite4.3: no detectable RNA, not accessible) and two unmethylated sites (MCsite4.4: no detectable RNA, accessible; and MCsite4.7: no detectable RNA, accessible). The DNA methylation patterns were complex, encompassing all three types of 5mC at MCsite4.1 and MCsite4.3 (Figure 7b). Specifically, there were three hypermethylated 5mCs at the MCsite4.1‐CRISPR and MCsite4.3‐CRISPR targeting sites, respectively, with three and four 5mCs on the left and right arms of MCsite4.1‐TALEN, and two and four 5mCs on the left and right binding sites of MCsite4.3‐TALEN, respectively. In contrast, complete demethylation was observed at MCsite4.4 and MCsite4.7.

To evaluate the expression and editing efficiency of CRISPR/Cas9, we utilized a two‐gRNA expression cassette. One gRNA targeted *CHLI2* serving as a positive control, and the other gRNA targeted MCsite4‐CRISPR sites (Figure S9). Efficient genome editing was observed at hypomethylation sites, specifically MCsite4.4 and MCsite4.7, using CRISPR/Cas9 and TALEN but not ngTALEN (Figure 7c,d). Consistent with editing results at the *FWA* locus, the performance of TALEN was robust at the less methylated sites (Figure S10A). The co‐editing frequency of the positive control *CHLI2* and MCsite4‐CRISPR by CRISPR/Cas9 was also quite high with more than 50% total editing efficiencies. qRT‐PCR measurements of the expression levels of the editors showed that CRISPR/Cas9 expressed at surprisingly higher levels than TALEN (10.8‐fold) and ngTALEN (6.2‐fold) (Figure S10B). Given that ngTALEN does not bind to non‐methylated base C, numerous mismatches occurred at these unmethylated sites, leading to low editing efficiency for ngTALEN at MCsite4.4 (10%) and no editing at MCsite4.7. Conversely, neither CRISPR/Cas9 nor TALEN/ngTALEN produced detectable genome editing at highly methylated sites, MCsite4.1 and MCsite4.3 (Figure S10C). Overall, the corresponding DNA molecules in the genome carry a mixture of 5mCs and other chromatin features in the target binding regions, making them suboptimal targets for these editors.

### Structural basis for recognition of NG‐RVD with 5mC

The direct binding of ngTALEN to 5mCs, along with its significantly stronger binding affinity compared with TALEN for methylated DNA, has been clearly demonstrated in previous *in vitro* binding assays. This finding has been further validated by solving the crystal structures of the ngTALEN–DNA complex (Deng et al., 2012). To provide additional evidence supporting the enhanced binding of ngTALEN to methylated DNA compared with TALEN, we utilized AlphaFold3 modeling (Abramson et al., 2024). The TALEN protein residues and their respective DNA‐binding sequences were uploaded to the AlphaFold3 Server (https://alphafoldserver.com/). When the DNA is methylated, a methylation modification mark was added to the DNA sequence. To illustrate chromatin features beyond 5mC modifications that inhibit ngTALEN editing, we analyzed editors targeting MCsite4 as an example. We obtained the structures of the TALEN–DNA complexes, which included the following: specific RVD HD opposite base C, non‐specific RVD HD opposite 5mC (designated MCsite4‐TALEN), non‐specific RVD‐NG opposite C, and specific RVD‐NG opposite 5mC (designated MCsite4‐ngTALEN).

**Figure 8:**
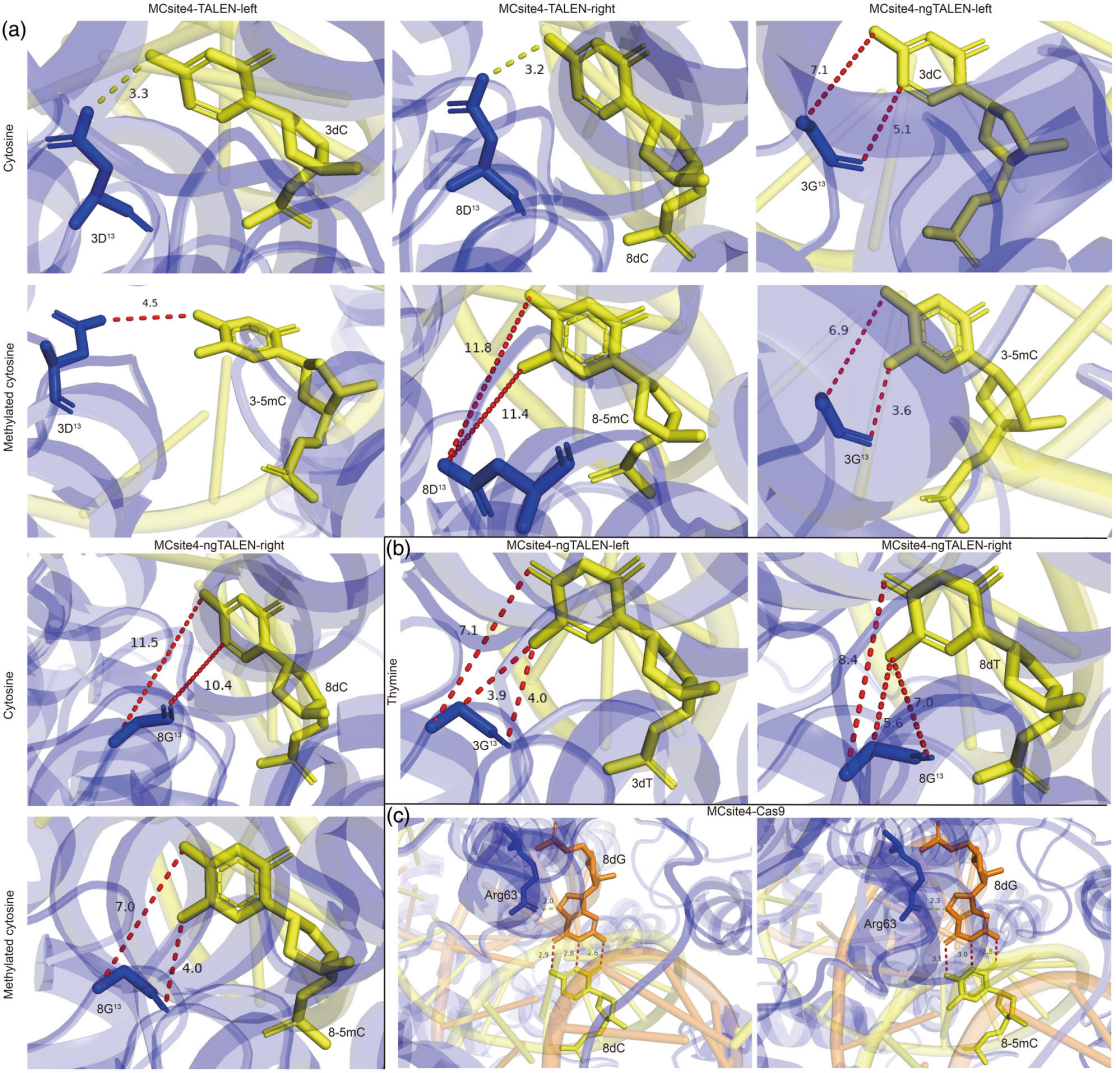
Comparison of interaction strength between editor proteins and the corresponding DNA targets at MCsite4 loci. (a) The carboxylate oxygen atom of Asp13 in the left arm of TALEN repeat 3 accepts a hydrogen bond (yellow dashed lines) from the amine group of cytosine. The positioning of Gly13 in the left arm of ngTALEN repeat 3 provides sufficient space to accommodate the 5‐methyl group of 5mC (red dashed lines indicate the distances). The carboxylate oxygen atom of Asp13 in the right arm of TALEN repeat 8 accepts a hydrogen bond from the amine group of cytosine. The placement of Gly13 in the right arm of ngTALEN repeat 8 allows sufficient space to accommodate the 5‐methyl group of 5mC. (b) RVD‐NG opposite thymine (T) serves as a control. (c) The Arg63 residue of the Cas9 protein forms a hydrogen bond with 8dG in the sgRNA–DNA duplex.

Each TALEN repeat comprises two helices connected by an RVD loop, which extends into the major groove of DNA and interacts with target bases. All TALEN repeats exhibit highly similar two‐helix conformations. The specific distinctions in the recognition of the first 5mC by TALENs are presented (Figure 8a,b). The MCsite4‐TALEN‐L repeat 3, with RVD HD (3D^13^), forms a hydrogen bond with the cytosine at position 3 (3dC) on the target DNA. In contrast, the distance between 3D^13^ and the first methylated cytosine at position 3 (3‐5mC) is 4.5 Å, which is too large to form optimal van der Waals interactions. However, the short side chain of Gly13 provides sufficient space to accommodate the 5‐methyl group of 5mC. The distances between these groups are shorter for MCsite4‐ngTALEN‐L opposite 5mC (3.6 Å) than for MCsite4‐ngTALEN‐L opposite base C (5.1 Å). This may enable the NG‐RVD to form strong non‐polar van der Waals interactions with the 5‐methyl group of 5mC. Similarly, for the right TALEN arms and interacting DNA targets, the HD‐containing repeat 8 at the RVD loop (8D^13^) forms a hydrogen bond with the base C at position 8 (8dC), while the distance for HD‐5mC is 11.8 Å, indicating that no direct interaction exists. The distance between NG in the loop of the 8th TALEN repeat (8G^13^) and the first 5mC in the target DNA (8dC) is 10.4 Å, whereas it is 4.0 Å for the corresponding NG‐5mC, where strong van der Waals interactions exist. This provides the structural basis for the specific recognition of RVD‐NG with 5mC. For the recognition of the Cas9 protein with the sgRNA–DNA duplex, the interaction is the strongest and there are no observable distinctions between unmethylated (ipTM = 0.88) and methylated DNA (ipTM = 0.89) (Figure 8c). This observation corroborates previous publications indicating that methylation does not interfere with the recognition of Cas9 and target DNA but does affect the enzymatic cleavage of the target (Das, 2021). In line with the above observations, the overall predicted ipTM score (interface predicted template modeling score) is higher for the recognition of NG‐5mC (0.88) than for NG‐C (0.7), HD‐C (0.73), and HD‐5mC (0.77) (Table S1). Together, the results of this structural modeling support the specific interaction of NG‐5mC, thereby demonstrating that chromatin features other than 5mC limit ngTALEN editing at sites with complex epigenetic modifications.

Finally, to demonstrate the broader applicability of ngTALEN in editing highly methylated sites, we targeted TALEN and ngTALEN editors to MCsite5 (Weiss et al., 2022), which includes two heavily methylated sites and one less methylated site (Figure S11A). As previously reported for editing at the *FWA* locus, ngTALEN improved editing efficacy at highly methylated sites compared with TALEN (Figure S11B), increasing from 3% with TALEN to 23% with ngTALEN (Figure S11C). Therefore, ngTALEN has broad applicability for enhancing editing at sites with multiple 5mC modifications.

## DISCUSSION

Cytosine methylation is commonly found in the genomes of plants and vertebrates (Kaya et al., 2017). However, the affinity of the TALEN‐cytosine‐recognition module for 5mC is low (Valton et al., 2012). The binding of CRISPR/Cas9 to its targets is also influenced by the chromatin contexts (Kuscu et al., 2014), suggesting the crucial role of epigenome state in efficient genome editing. To overcome the inhibition of editing efficiency caused by methylation, we utilized ngTALEN, which has demonstrated a strong binding affinity to 5mC through *in vitro* chemical binding assays and crystal structure determination, to recognize 5mC at methylated sites in plants. To disentangle the potential influence of distinct DNA sequence composition and methylation status, we directed the ngTALEN editor to the *FWA* promoter, a common target for studies related to epigenetic modification, placing the editors within an overlapping target window in both the Col‐0 and *fwa* epimutant backgrounds. To compare editing efficiency at sites with varying methylation statuses, we targeted the editors to three sites within the *FWA* promoter. Additionally, as methylation at the promoter and gene body has distinct functions, we defined, for the first time to our knowledge, two methylated sites corresponding to the CDS regions of exons 6 and 8, and targeted the genome editors to them as well. To demonstrate that TALEN editors are robust in addressing various sites, we compared the frequently used CRISPR/Cas9 system with TALEN and ngTALEN editors. Finally, to test the broader applicability and potential limitations of ngTALEN, we directed the editors to more challenging targets, MCsite4 and MCsite5, which contain multiple epigenetic modifications predominantly located within intergenic regions. We found that ngTALEN is robust for editing multiple CG‐methylated sites. Editing at target DNA with one or two CG methylations was less affected than editing at multiple 5mCs. Therefore, the enhanced efficiency of ngTALEN editing was greater at the highly methylated promoter regions compared with the less methylated gene body of *FWA*. However, sites with modifications in addition to CG methylation may present challenges for ngTALEN.

### Editing by CRISPR/Cas9 and TALEN

There is a growing focus on the effect of heterochromatic features on mutagenesis efficiency in multiple eukaryotic systems (Daer et al., 2017). However, methods to manipulate chromatin features to enhance genome editing in plants remain limited. TALEN is the first easy‐to‐use genome editing device applicable in multiple species. Its flexibility and precise positioning are unmatched and have continued to evolve with new functionalities. Although TALEN‐based genome editing tools are now much less used in comparison to the vast CRISPR repertoire, they share key features like specificity and efficiency. Consistent with this, the present study revealed comparable editing efficiency for TALEN and CRISPR crossing diverse sites. Moreover, the pure protein nature of TALEN and its flexible positioning are unprecedented. With advancements in building techniques, TALEN‐based editors continue to expand, diversify, and become more potent. Indeed, a recent advance uses a single protein called a compact TALEN (cTALEN) for genome editing, thus greatly simplifying the TALEN vector assembly (Beurdeley et al., 2013; Kazama et al., 2019). Despite the binding of TALEN to target sites being inhibited by chromatin structures, TALEN is less compromised by chromatin than CRISPR/Cas9 due to its higher activity in heterochromatic regions of the chromosome (Becker & Boch, 2021). Furthermore, CRISPR/Cas9‐induced mutations are typically small deletions or 1‐bp insertions (Weiss et al., 2022), whereas TALEN usually favors the formation of larger deletions at the target site as a result of imperfect repair by non‐homologous end joining (NHEJ) (Becker & Boch, 2021). Similar editing outcomes have been shown in the present study.

Since the sequences in the target window contribute to editing efficiency (Liu, Li, et al., 2019), we targeted six different sequences within one gene locus: three in the promoter region and the other three in the gene body of *FWA*. This strategy aimed to exclude the effect of sequence bias on genome editing efficiency and confirmed that 5mC inhibits editing and TALEN performs comparably to CRISPR/Cas9 in GC‐methylated regions. Different from the previous report stating that high levels of promoter methylation, but not gene body methylation, decreased the frequency of CRISPR/Cas9‐mediated mutagenesis (Přibylová et al., 2022), we found that methylation in the gene body, specifically in the CDS regions of the *FWA* locus, also impeded editing for both CRISPR/Cas9 and TALEN. This discrepancy may be due to random insertions of the GFP reporter in multiple sites across the genome in *Nicotiana benthamiana*, as well as differences in local chromatin contexts around the targeting sites. Factors other than 5mC in the former study might have obscured the differences.

Notably, the DNA base T is similar in chemical structure to 5mC with the only difference occurring at position 4, which is not involved in binding to TALE repeats. An *in vitro* binding assay revealed that 5mC can be recognized by NG in RVD of TALEN (Deng et al., 2012). This is plausible because the lack of a side chain in Gly^13^ not only provides sufficient space to accommodate the 5‐methyl group of 5mC but also allows optimal van der Waals interactions to occur between the Cα atom of Gly^13^ and the 5‐methyl group. In this study, we developed methods using ngTALEN to recognize 5mC at targeting loci, with the aim of enhancing editing efficiency. We concluded that ngTALEN provides a robust method for enhancing genome editing efficiency at sites containing 5mCs in *Arabidopsis*.

The pipeline developed in the present study for screening sgRNAs is of high efficiency. The U6‐26 promoter from *A*. *thaliana*, which is optimized for expression in this plant, was utilized in this study to drive the expression of gRNAs. Since multiple “T” nucleotides in the tracrRNA and in spacer positions 17–20, when paired with the Hsu tracrRNA, lead to lower sgRNA expression (DeWeirdt et al., 2022), all of the sgRNAs selected through screening using the developed pipeline in this study do not contain multiple “T” nucleotides at spacer positions 17–20. Additionally, the Doench Rule Set 3 score evaluation system was developed using mammalian cells, which may differ from plant systems. The score ranges from −200 to 200, which is quite broad; a score of −10 may not necessarily indicate low activity of an sgRNA. Therefore, we did not include this parameter in our pipeline. For example, the cdsFWA‐CRISPR2 sgRNA, with a Doench Rule Set 3 score of −10, was predicted to be incompatible with the U6/U3 promoter but achieved an editing efficiency of approximately 50% in cdsFWA‐CRISPR2 in *fwa* (Figure 3c), indicating effective expression of sgRNA driven by the U6‐26 promoter. Similarly, the sgRNA for MCsite4‐CRISPR does not contain multiple “T” nucleotides at spacer positions 17–20, achieving more than 60% editing frequency at less methylated sites in this study and in a previous study by another group (Weiss et al., 2022). Additionally, two other software programs for sgRNA evaluation, CRISPR‐RGEN and inDelphi, were utilized to assess sgRNAs, yielding scores of 63.9 (CRISPR‐RGEN) and 41.0 (inDelphi) for the cdsFWA‐CRISPR2 sgRNA, and 55.0 (CRISPR‐RGEN) and 43.0 (inDelphi) for the MCsite4‐CRISPR sgRNA, respectively.

### DNA methylation and its impact on genome editing

Several tools have been developed to predict the efficiency and mutation outcomes solely based on targeted sequences (Weiss et al., 2022), including CRISPOR and TALEN Targeter, which were used for prediction in this study. The chromatin accessibility in the targeting regions has an impact on the binding ability of TALEs (Erkes et al., 2021). Furthermore, methylation of cytosine *in vivo* may render the DNA sequence unfit for binding by the designed genome editors (Deng et al., 2012). Therefore, the *in vivo* methylation status on the target sequence must be considered when designing specific DNA‐binding sequences. Fortunately, a tool for this purpose is already available: EpiTALE (https://jstacs.de/index.php/EpiTALE) (Erkes et al., 2021).

In Col‐0, expression of *FWA* is established through maternal gametophyte‐specific gene activation before double fertilization, and the expression of *FWA* in the endosperm does not contribute to the next generation (Kinoshita et al., 2004). The maintenance of endosperm‐specific and parent of origin‐specific *FWA* expression depends on Methyltransferase 1 (MET1)‐mediated CG methylation (Kinoshita et al., 2004). When methylation is lost in the embryonic lineages due to a *met1* mutation, the *fwa* epigenetic mutation and the associated late‐flowering phenotype are stably inherited over many generations. To rule out the potential influence of other chromatin states, aside from CG methylation, on editing efficiency, we analyzed the chromatin states at the *FWA* locus using the AraENCODE database. Since the chromatin state datasets, including H3K4me3, H3K27ac, H3K9ac, RNA Pol II ChIP‐Seq, H3K4me1, H3K27me3, and H3K9me2, are not available for the *fwa* epimutant, we compared datasets from wild‐type Col‐0 and the *met1‐3* mutant. The *FWA* locus was highly methylated in Col‐0 (Figure S12A) but showed demethylation in the *met1‐3* mutant (Figure S12B). Compared with Col‐0, the *FWA* transcripts along with the epigenetic marks H3K4me3, H3K27ac, and H3K9ac were highly represented in the *met1‐3* mutant, while other chromatin features, including chromatin accessibility, H3K4me1, H3K27me3, and H3K9me2, remained almost unchanged between the two backgrounds. This observation is consistent with our results indicating that the demethylation‐induced reactivation of *FWA* in the *fwa* epimutant is closely associated with activation marks on the chromatin, leading to altered flowering time. Additionally, targeted demethylation of the *FWA* promoter in Col‐0 generated demethylated *fwa* epimutants with delayed flowering times, suggesting that CG methylation is the primary cause of the altered phenotype (Gallego‐Bartolomé et al., 2018). Together, these findings indicate that CG methylation is a major factor affecting genome editing efficiency at the *FWA* locus. In this study, we used the *RPS5A* promoter to drive the expression of the editors. This promoter has been reported to drive high levels of gene expression in egg cells and early embryos (Tsutsui & Higashiyama, 2017). The early expression of editors driven by the *RPS5A* promoter may have contributed to the homozygous editing in many T_1_ transformants or T_0_ regenerants, especially for TALEN and ngTALEN editors. In cases where transient expression of *FWA* in the gametophyte and demethylation in the *FWA* promoter occur during floral‐dipping transformation of Col‐0, we also performed tissue culture‐mediated transformation to introduce the editors into plant cells. Fortunately, we observed differentiated editing frequencies from the materials generated by both of these methods.

The genome‐wide DNA methylomes of Col‐0 during the tissue culture of the leaf‐to‐callus transition were largely unchanged (Shim et al., 2021). Compared with regenerable calli, the promoter of *FWA* is demethylated and highly expressed in non‐regenerable calli (Dai et al., 2021). Similarly, explants from the epimutant *fwa* exhibit strong expression of *FWA* and reduced regenerability compared with the wild‐type. Bisulfite sequencing of the two direct repeats in the *FWA* promoter revealed 86% CG methylation from Col‐0 regenerated tissues, while only 0.4% CG methylation was detected in the *fwa* epimutant. Consistent with this, the expression of *FWA* was suppressed in Col‐0 explants, whereas it was abundant in *fwa* explants (Dai et al., 2021). The inhibition of regeneration by *FWA* is mediated by the direct repression *of WUSCHEL‐RELATED HOMEOBOX 9* (*WOX9*), which is necessary for shoot apical meristem formation. Consistent with these observations, we noted similar callus induction for Col‐0 and the *fwa* epimutant. However, the regeneration of embryogenic calli and regenerated plants was far less in the *fwa* epimutant background. Therefore, the regeneration capacity, along with the methylation status and expression of *FWA*, is tightly connected during the tissue culture process.

One of the advantages of TALEN towards CRISPR/Cas9 lies in the flexible design of its binding specificity. It is necessary to find a suitable PAM that allows placing CRISPR/Cas9 such that the target base is within the editing window. If other CRISPR‐based editors are used with loose PAM selection and a broad editing window, multiple bases within the editing window can be edited, potentially causing unwanted amino acid changes (Anzalone et al., 2020). In contrast, TALEN can be positioned freely, and both its length and the length of the editing window can be adjusted within a wide range, ensuring unprecedented accuracy in positioning and flexibility (Becker & Boch, 2021). Due to the flexibility of TALEN target positioning, 5mC sites can be avoided by sliding the TALEN recognition targets a few bases upstream or downstream, using different sizes of target windows, or optimizing the sequences linking the TALE and FokI (Tan et al., 2019). Therefore, we first fixed the overlapping editing window by CRISPR/Cas9 and then designed TALEN arms flanking the editing window. However, this design inevitably resulted in a higher predicted editing efficiency for CRISPR/Cas9 compared with TALEN, based on the sequences binding to DNA.

### The outcomes of genome editing by TALEN and ngTALEN

The *Arabidopsis FWA* gene encodes an HD‐ZIP IV homeodomain protein that binds to TAAATG motifs in the promoters of its target genes (Dai et al., 2021). The promoter of *FWA* contains two direct repeats that are heavily methylated in Col‐0, while demethylation of this region reactivates transcription of *FWA* in *fwa* epimutant (Fujimoto et al., 2008). This epimutant *fwa* displays a strong late‐flowering phenotype due to the ectopic expression of *FWA*, which interacts with and suppresses FT to delay flowering. Plants overexpressing a truncated C‐terminal interaction domain of FWA do not exhibit a late‐flowering phenotype, suggesting that the C‐terminal interaction domain of FWA is crucial for this phenotype (Ikeda et al., 2007).

We obtained mutants by TALEN and ngTALEN targeting the C‐terminus of *FWA*. Some of these mutants reversed the late‐flowering phenotype of *fwa*, indicating truncation of the FWA protein at the C‐terminus and disruption of its interaction with FT. Direct organogenesis bypasses callus formation and directly induces the formation of roots, shoots, and leaves from explants. This method has been applied in many species, such as the model plant *A. thaliana* and the ornamental plant *Oxalis vulcanicola* “Sunset Velvet” (Dai et al., 2021; Wang et al., 2025). In wild‐type plants, hypermethylation of the *FWA* promoter represses *FWA* expression, leading to higher expression of *WUSCHEL‐RELATED HOMEOBOX 9* (*WOX9*) and the promotion of direct shoot regeneration *in vitro*. In contrast, in the *fwa* epimutant, hypomethylation of the *FWA* promoter results in higher expression of this gene, leading to repressed *WOX9* expression and direct shoot regeneration *in vitro* (Dai et al., 2021). In this study, editor transformants targeting the promoter regions of *FWA* barely yielded any regenerants, suggesting that alteration of *FWA* expression by genome editing suppressed *WOX9* expression and, consequently, plant regeneration.

### Other chromatin features and their impact on genome editing

In plants, DNA methylation occurs in various cytosine contexts, specifically CG, CHG, and CHH (where H is A, T, or C), and is controlled by different DNA methyltransferases. Among these enzymes, MET1 is primarily responsible for maintaining symmetric methylation in the CG context (Gallego‐Bartolomé et al., 2018). The silencing of the *FWA* gene is dependent on cytosine methylation, as it becomes derepressed when *MET1* function is lost (Fujimoto et al., 2008). This observation aligns well with our findings that methylation at the *FWA* locus is predominantly CG methylation.

Importantly, some of the MCsite4 loci are located in heterochromatin (i.e., MCsite4.1), which is highly enriched with all three types of DNA methylation and other chromatin features such as H3K9me2 histone methylation (Weiss et al., 2022). This may represent a more challenging environment for targeted editing. By contrast, at hypomethylation sites MCsite4.4 and MCsite4.7, comparable editing efficiency was observed for MCsite4‐TALEN compared with MCsite4‐CRISPR, consistent with findings on *FWA* editing. However, since ngTALEN does not bind to unmethylated cytosines, mismatches occurred at MCsite4.4 and MCsite4.7, thereby completely inhibiting editing. Furthermore, the editing was completely blocked by complicated chromatin features at MCsite4.1 and MCsite4.3 for all three editors.

Our method has several advantages over others when analyzing the effect of methylation on genome editing efficiency. For example, we simplified the editing comparison by fixing the DNA sequence at the *FWA* locus, which primarily contains CG methylation. The corresponding target sites in Col‐0 and *fwa* share the same sequence but exhibit contrasting CG methylation statuses. This design allowed us to not only confirm previous observations that DNA methylation and chromatin contexts inhibit editing but also make new discoveries. Specifically, we found that methylation within the gene body of *FWA* impeded editing for both CRISPR/Cas9 and TALEN, especially in editor transformants generated through tissue culture‐mediated transformation. Nonetheless, our method also has some limitations. For instance, ngTALEN does not recognize non‐methylated cytosine very well (Deng et al., 2012). If the methylation status is unclear at a specific targeting site, recognition of multiple cytosines by ngTALEN might introduce mismatches, thereby limiting the frequency of editing. However, this side effect can be mitigated by using a universal RVD that recognizes both C and 5mC. Additionally, factors other than 5mC, such as chromatin structures and histone methylation, can affect editing, and these cannot be addressed by ngTALEN alone. Closed chromatin inhibits CRISPR/Cas9 binding and editing at specific target sites, while artificial reversal of the silenced state restores editing efficiency in a transgenic cell line (Uusi‐Mäkelä et al., 2018). Furthermore, nucleosome‐occupied regions prevent Cas9 binding (Daer et al., 2017). By utilizing Cas9‐TV/dsgRNA (which fuses a synthetic transcription activation domain TV to Cas9 and combines a proximally binding dead dsgRNA), improved editing efficiency of several folds was achieved in both open and closed chromatin regions in rice (Liu, Yin, et al., 2019). Infusion of ngTALEN with factors such as the TV domain may facilitate the editing of sites with DNA methylation and other chromatin features.

Although the assembly of TALEN pairs increased the cloning effort, the reduced constraint in selecting target sites for TALEN and the simultaneous binding of both arms to neighboring target sites (covering 12–42 bp) to induce a DSB by FokI decrease the chance for off‐target effects in comparison to the CRISPR/Cas9 system (Becker & Boch, 2021; Kaya et al., 2017). Moreover, our previous data suggest that TALE‐based editors do not introduce a significant number of off‐target mutations and show no discernible correlation between the number of similar sequences and the frequency of off‐target mutations (Hosoda et al., 2024). Null‐segregant plants that carry the targeted homozygous mutations without off‐targets have been obtained in later generations as well. Yet, a genome‐wide systematic comparison of the off‐target activities between TALEN and CRISPR/Cas9 is still to be investigated. Overall, the present study demonstrated that the editing was inhibited by 5mC at both the promoter and gene body of *FWA* locus for both CRISPR/Cas9 and TALEN. Moreover, TALEN is robust and performed comparable to CRISPR/Cas9 across various sites, while ngTALEN performed the best among the three editors at the hypermethylated promoter of *FWA*. Therefore, we have developed ngTALEN as a robust tool for enhancing editing at sites with various numbers of 5mC modifications.

## MATERIALS AND METHODS

### Plant materials and growth conditions

Wild‐type *Arabidopsis thaliana* ecotype Columbia‐0 (Col‐0) and the *fwa‐d* epimutant in Col‐0 background (Kinoshita et al., 2007) (a kind gift from Dr. Tetsuji Kakutani, the University of Tokyo) were used in this study. The seeds were sown and germinated on half‐strength Murashige and Skoog solid medium (1/2 MS). Three‐week‐old plantlets were transplanted to Jiffy‐7® pots (Jiffy, http://www.jiffypot.com) and grown under conditions of 22°C, with a light intensity of 50–150 μmol m^−2^ sec^−1^ and a 16‐h light/8‐h dark photoperiod in a culture room. The T_2_ and T_3_ progenies were produced by self‐crossing. Flowering time was measured by counting the number of rosette leaves prior to bolting.

### Vector construction

The backbone of the binary destination vectors utilized in this study contains the *Arabidopsis RPS5A* promoter, *SV40NLS*, and *HSP* terminator (Addgene, Watertown, MA, USA; #193370). The pair of right and left TALEN arms targeting a specific sequence were constructed using a Platinum Gate TALEN Kit provided by Addgene (Addgene#1000000043) and assembled into a single Ti‐plasmid *via* a Gateway LR reaction (Thermo Fisher Scientific, Waltham, MA, USA) as described in our previous study (Arimura, 2022; Nakazato & Arimura, 2025). The CRISPR/Cas9 vector was constructed according to a previous publication (Vad‐Nielsen et al., 2016). The backbone vector, derived from pDESTpK7WGS2‐pRPS5A‐NLSCas9‐OleGFP, already includes a Cas9 expression cassette driven by the *RPS5A* promoter and an *Oleosin 1* (*Ole1*) promoter specifically driving GFP expression, which served as a selection marker for transgenic seeds. The customized DNA oligonucleotides were synthesized by Sigma Aldrich (Tokyo, Japan), annealed, and inserted into the entry vector pEnChimera between two *Bbs*I sites (Fauser et al., 2014). If a second sgRNA is needed, the entry vector was cleaved by *Xba*I at the site flanking the first sgRNA sequence, and the PCR‐amplified second sgRNA expression cassette was inserted there through the In‐Fusion cloning system (Clontech, Mountain View, CA, USA). Finally, the sgRNA expression cassettes in pEnChimera were ligated into the destination vector containing Cas9 *via* a Gateway LR reaction using LR Clonase II Plus enzyme (Thermo Fisher Scientific, Waltham, MA) as described in our previous study (Nakazato et al., 2021). The expression of sgRNAs are driven by *A. thaliana* derived U6‐26 promoter. The primers used are listed in Table S2. The Ti‐plasmids were introduced into *Agrobacterium tumefaciens* strain C58C1 (pMP90) by electroporation. The introduction of T‐DNA from the Ti‐plasmids into the nucleus was achieved through *Agrobacterium*‐mediated root‐explant transformation (Valvekens et al., 1988) or floral‐dipping methods (Zhang et al., 2006) in *Arabidopsis*. Regenerated plants from kanamycin selection or plantlets from germinated GFP‐positive seeds were verified by amplicon Sanger sequencing.

### Tissue culture‐mediated transformation

The tissue culture‐mediated transformation of *Arabidopsis* roots and plant regeneration was performed following a standard protocol (Valvekens et al., 1988). Briefly, sterilized seeds of *A. thaliana* were placed on MS medium supplemented with 1 × B5 vitamin (prepared from a 1000 × stock solution; Wako Pure Chemical Industries, Tokyo, Japan) and 20 g/L sucrose. These seeds were then cultured at 25°C in a light incubator with a 16‐h light/8‐h dark photo period and a light intensity of 60 μmol m^−2^ sec^−1^ for 12 days. Subsequently, the roots were cut and placed on callus induction medium (approximately 50 root segments per plate) containing 3.3 g/L B5 medium mixed salt (Wako Pure Chemical Industries), 1 × B5 vitamin (prepared from a 1000 × stock solution), 20 g/L glucose, 0.5 g/L 2‐(N‐morpholino) ethanesulfonic acid (MES), 0.5 mg/L 2,4‐D, and 0.05 mg/L kinetin. The root explants were maintained on this medium for 4 days. Next, the root explants were immersed in an *Agrobacterium* solution (OD_600_ = 0.1) with gentle agitation for 20 min. Following immersion, the explants were placed on sterilized filter paper to remove excess *Agrobacterium* and then transferred back onto callus induction medium for another 3 days. After this period, the root explants were transferred to shoot induction medium containing 3.3 g/L B5 medium mixed salt (Wako Pure Chemical Industries), 1 × B5 vitamin, 20 g/L glucose, 0.5 g/L MES, 5.0 mg/L 2iP, 0.15 mg/L IAA, 25 mg/L kanamycin, and 12.5 mg/L meropenem to induce shoot formation. Calli were induced from the root explants, and embryogenic calli formed after 2 months.

### DNA/RNA extraction and PCR analysis

Total DNA was extracted from young leaves of 2‐week‐old seedlings using the Maxwell RSC Plant DNA Kit (Promega, Madison, WI, USA; http://www.promega.com). Multiplex PCR was conducted with Quick Taq HS DyeMix (TOYOBO, Osaka, Japan) employing the primer combinations detailed in Table S2. The extracted total DNA from leaves of 2‐week‐old seedlings served as the template for PCR. The PCR products varied in length by at least 150 bp to facilitate their separation on an agarose gel. Additionally, total RNA was isolated from young leaves of 2‐week‐old seedlings with the ISOSPIN Plant RNA Kit (310‐08171; Nippon Gene, Toyama, Japan) and subsequently treated with RNase‐free DNAase I according to the manufacturer's instructions. Quantitative Real‐Time PCR (qRT‐PCR) was performed to quantify transcript levels using the One Step TB Green® PrimeScript™ RT‐PCR Kit II (Perfect Real Time, RR086B; TaKaRa, Shiga, Japan). *PEX4* (AT5G25760) was utilized as the internal control, and the 2^−▵▵Ct^ was applied to compare relative expression levels (Czechowski et al., 2005). The gene‐specific primer sequences employed are listed in Table S2. Each sample included at least three biological replicates and three technical replicates.

### Amplicon sanger sequencing

GFP‐positive seeds were selected under a fluorescent microscope using a seed picker. T_1_ plants were grown on 1/2 MS medium plates supplemented with 10 g/L sucrose and 125 mg/L claforan. For each transformed material, approximately 30 plants were subdivided into three groups of 10 plants each, and genomic DNA was isolated using a simplified isolation buffer (100 mm Tris·HCl, pH9.5; 10 mm EDTA, pH8.0). One rosette leaf from a 2‐week‐old seedling was detached and placed into 50 μl of isolation buffer in a 200 μl tube. DNA isolation was performed using a PCR thermal cycler with a program of 95°C for 15 min. 0.5 μl of the isolated DNA was used as the template for PCR amplification (KOD One™ PCR Master Mix‐Blue, KMM‐201; TOYOBO). A 619 bp fragment of the *FWA* promoter, encompassing the two direct repeat regions, was amplified using primers listed in Table S2. Additionally, a 985 bp DNA fragment of *FWA* spanning the exon6 to exon8 coding region was amplified and sequenced to analyze editing. About 3 μl of the PCR products were analyzed by agarose gel electrophoresis, and the remaining products were subjected to purification using a DNA Extraction from Agarose Gel/Purification of PCR Products Kit (FG91302; NIPPON Genetics, Tokyo, Japan). About 60 ng of the purified PCR product were sent for Sanger sequencing (Eurofins Genomics, Tokyo, Japan). Data analysis was performed using the Geneious Prime v.2023.2.1 software (http://www.geneious.com).

### Bisulfite sequencing

Genomic DNA was extracted from the first true leaves of 13‐day‐old plantlets using the Maxwell® RSC Plant DNA Kit (Promega). A quantity of 200 ng of DNA was treated with the EZ DNA Methylation‐Lightning™ Kit (Zymo Research, Irvine, CA, USA) following the provided protocols, and the modified DNA was used as a template for PCR amplification. A 391 bp fragment of the *FWA* promoter, covering the two tandem repeat regions, was amplified using the primers listed in Table S2. Four individual plantlets were sequenced for each sample (*n* = 4).

### Data analysis

Plant images were captured using a Sony α6400 camera (SONY, Tokyo, Japan) and Leica MC170 HD (Leica, Wetzlar, Germany). Gel images were obtained with the ChemiDoc MP Imaging System (BIO‐RAD, Hercules, CA, USA). The images were processed and organized using Adobe Illustrator 2021. qRT‐PCR was performed on StepOnePlus Real‐Time PCR System (Thermo Fisher Scientific). GFP‐positive seeds were selected under a fluorescence microscope using a seed picker and germinated on 1/2MS medium. Approximately, 30 T_1_ plants for each construct were randomly divided into three subgroups, serving as repeats, with about 10 plants per repeat. The targeted CDS regions of T_1_ plants were amplified and Sanger sequenced. Across all the target regions, each editor generated mutations, and multiple T_1_ lines exhibited homozygous editing events. We calculated the total editing and homozygous editing frequencies for each repeat sample for all editor transformants in the Col‐0 or *fwa* epimutant backgrounds. Statistical data were analyzed and figures were generated using GraphPad Prism 8 software (La Jolla, CA, USA). The statistical significance of differences (*P < 0.05) was determined using a one‐way ANOVA multiple comparison test, with a minimum of three replicates per construct.

## AUTHOR CONTRIBUTIONS

SA conceived the project. SA and ZW designed the experiments. ZW constructed the CRISPR/Cas9 vectors and YT constructed the TALEN/ngTALEN vectors. ZW performed most of the experiments including the transformations, DNA and RNA extractions, amplicon PCR, multiplex PCR, qRT‐PCR, Sanger sequencing, alphafold3 modeling, and chromatin state analysis. MH helped with CRISPR/Cas9 vector construction and GFP‐positive seeds selection. AY helped with the TALEN/ngTALEN vector construction. IN and YT conducted bisulfite sequencing. IN provided technical suggestion on plant culture, RNA extraction, and qRT‐PCR analysis. ZW analyzed the data and drafted the manuscript. All authors read and approved of its content.

## ACCESSION NUMBERS

Whole genome bisulfite sequencing for *fwa* (GEO accession no. GSM2932309), *FWA* (AT4G25530), *ACTIN2* (AT3G18780), *PEX4* (AT5G25760), *CHLI2* (AT5G45930), MCsite4.1 (AT2TE16230), MCsite4.3 (AT2TE00030), MCsite4.4 (AT2TE00095), MCsite4.7 (AT4TE51005), MCsite5.1 (AT1G23915), MCsite5.3 (AT4G20960), and MCsite5.4 (AT4G09380).

## CONFLICT OF INTEREST

All authors declare that they have no competing interests.

## Supporting information


**Figure S1.** Target design and evaluation.
**Figure S2.** TALEN vector assembly.
**Figure S3.** Induction of embryogenic calli for the editors targeting the promoter regions of *FWA* locus.
**Figure S4.** Comparison of the editing efficiency of editors on less methylated CDS regions of *FWA*.
**Figure S5.** Comparison of editing efficiency on highly methylated promoter regions of *FWA*.
**Figure S6.** Flowering phenotype of the edited offspring in T_2_ and T_3_ generations.
**Figure S7.** TALEN and ngTALEN editing of the *FWA* promoter leads to the heritable reversal of the *fwa* late‐flowering phenotype in the T_3_ generation.
**Figure S8.** Comparison of editing efficiency of editors across various sites.
**Figure S9.** Two‐sgRNA vector construction.
**Figure S10.** Comparison of editing efficiency of editors targeting the MCsite4 loci.
**Figure S11.** Comparison of editing efficiency of editors targeting the MCsite5 loci.
**Figure S12.** Chromatin states at the *FWA* locus in Col‐0 and *met1‐3* plants.
**Table S1.** Alphafold3 modeling of the overall interaction.
**Table S2.** Primer list.

## Data Availability

All relevant data can be found within the article and supporting materials.
