# Supplementary material for: Using ngTALEN to improve genome editing efficiency on targets containing 5‐methylcytosines
Source: Plant J. 2026 Apr 4;126(1):e70826. doi: 10.1111/tpj.70826 (PMC13050242; doi:10.1111/tpj.70826)
Supplement: Supplementary file 1 — Figure S1. Target design and evaluation. Figure S2. TALEN vector assembly. Figure S3. Induction of embryogenic calli for the editors targeting the promoter regions of FWA locus. Figure S4. Comparison of the editing efficiency of editors on less methylated CDS regions of FWA. Figure S5. Comparison of editing efficiency on highly methylated promoter regions of FWA. Figure S6. Flowering phenotype of the edited offspring in T2 and T3 generations. Figure S7. TALEN and ngTALEN editing of the FWA promoter leads to the heritable reversal of the fwa late‐flowering phenotype in the T3 generation. Figure S8. Comparison of editing efficiency of editors across various sites. Figure S9. Two‐sgRNA vector construction. Figure S10. Comparison of editing efficiency of editors targeting the MCsite4 loci. Figure S11. Comparison of editing efficiency of editors targeting the MCsite5 loci. Figure S12. Chromatin states at the FWA locus in Col‐0 and met1‐3 plants. Table S1. Alphafold3 modeling of the overall interaction. Table S2. Primer list. [file TPJ-126-0-s001.docx]

**Figure S1.** Target design and evaluation. (A) Schematic illustration of the target sites showing the editing window, sgRNA binding sites, PAM sequence (bold, underlined), and paired TALEN binding sites at *FWA* locus. CRISPR/Cas9, TALEN and ngTALEN had overlapping editing windows with the same levels of 5mC. Three such overlapping editing targets in the *FWA* promoter representing hypermethylated regions and another three in the CDS region (corresponding to exon 6 and exon 8 coding regions) representing the less methylated regions on the *FWA* gene body in Col-0. Red C indicates hypermethylation. The editing window is framed with a rectangle. (B) Schematic pipeline used to select sgRNAs for CRISPR/Cas9 using the CRISPOR online software. Numbers in the box indicate the number of sgRNAs recalled. (C) Summary of the predicted editing scores for sgRNAs using CRISPOR and summary of the predicted editing scores for TALEN arms using TALEN Targeter.

**Figure S2.** TALEN vector assembly. (A) Schematic illustration of the assembly of TALEN expression vectors [Adapted from (Nakazato et al., 2021)] with cdsFWA-TALEN1L (left arm) as an example. The first two steps for assembling of a complete binding arm were conducted using the Platinum Gate TALEN Assembly Kit. The third step, for creating a pair of tandem expression arms, was performed using Multisite Gateway (Thermo Fisher Scientific). The left and right binding arms produced in the first two steps, along with an Entry vector 2 containing an *Arabidopsis* heat shock protein terminator and an *RPS5A* promoter, can be assembled into a destination Ti-plasmid using Multisite Gateway. Primary transformants can be selected based on the kanamycin resistance cassette (*NPTII*) for tissue-culture-mediated transformation or the proOle-eGFP expression cassette for floral-dipping-mediated transformation. The different domain structures are highlighted. (B) Cytosine methylation on the binding sites for the *FWA* locus and the ngTALEN arms, which use NG instead of HD to recognize 5mC. (C) Representative colony PCR showing the isolation of positive clones for the assembly of TALEN arms. (D) The final tandem expression Ti-plasmids were verified by *Nde*I and *Pvu*II enzyme digestion at multiple sites within the vector. (E) A summary of the *Nde*I and *Pvu*II enzyme digestion sites within the vector. (F) The distribution of *Nde*I and *Pvu*II enzyme digestion sites is represented on the vector map.

**Figure S3.** Induction of embryogenic calli for genome editors targeting the promoter regions of the *FWA* locus. (A) Representative images showing the induced embryogenic calli for genome editors targeting the promoter of the *FWA* locus. (B) Statistics relating to embryogenesis for the genome editor transformants in both Col-0 and *fwa* backgrounds after culturing for two months. Data represent means ± SD (n = 3). For each transformation, embryogenic calli generated from three plates, with each plate inoculated with approximately 50 root explants, were counted. (C) Summary of editing frequencies on target windows in regenerated T_0_ plantlets with Col-0 or *fwa* epimutant backgrounds. Abbreviations: homo, homozygous editing; h/c, heterozygous/chimera editing. (D) Comparison of editing frequencies between CRISPR/Cas9 and TALEN in the less methylated CDS regions of *FWA* in regenerated T_0_ plantlets. Editing events on the three targets were combined for analysis. Abbreviations: homo, homozygous editing; h/c, heterozygous/chimera editing.

**Figure S4.** Comparison of the editing efficiency of editors on less methylated CDS regions of *FWA*. (A) The sequence and location of target sites within CDS regions of *FWA* corresponding to exon 6-8 are depicted. Sequences of exon 6 and 8 are underlined. The sgRNA binding sites for CRISPR/Cas9 are highlighted in green, while 5mCs are marked in red. (B) A summary of violin plot presenting the combined analysis of total editing and homozygous editing frequencies across the three target sites in T_1_ transformants with Col-0 or *fwa* epimutant backgrounds. (C) A representative image of T_1_ transformants of cdsFWA-TALEN1 in Col and cdsFWA-TALEN1 in *fwa* grown on 1/2 MS plates for three weeks. Approximately 30 plants were randomly divided into three subgroups as repeats, with 10 plants per replicate, and subjected to Sanger sequencing. Yellow arrows indicated the phenotypic reversion of the *fwa* epimutant in cdsFWA-TALEN1 in *fwa* transformants. A zoomed-in image of one such revertant, exhibiting early bolting, is shown below. (D) Specific numbers of edited plants and editing frequencies within the CDS regions of *FWA* target windows in T_1_ plantlets with Col-0 or *fwa* epimutant backgrounds. (E) Combined analysis of editing frequencies across the three CDS targets of *FWA*, showing the total numbers of edited plants and editing frequencies for each group of editor transformants. For the violin plots, each data point represents the editing frequency for 10 plants, representing a repeat for a specific editor. Abbreviations: homo, homozygous editing; h/c, heterozygous/chimera editing. The middle-dashed line represents the mean, and the other dashed lines represent the interquartile ranges. *P* values were calculated by One-way ANOVA multiple comparison tests. **P* < 0.05, ***P* < 0.01, ****P* < 0.001, *****P* < 0.0001.

**Figure S5.** Comparison of editing efficiency on highly methylated promoter regions of *FWA*. (A) Sequence and location of target sites in the promoter regions of *FWA* locus. The two-direct-repeat regions are underlined. The sgRNA binding sites for CRISPR/Cas9 are labeled in green, while 5mCs are marked in red. (B) Summary violin plot illustrating the combined analysis of total editing and homozygous editing frequencies across three target sites in T_1_ transformants, with Col-0 or *fwa* epimutant background. (C, D) Representative image show T_1_ edits of proFWA-TALEN1 in *fwa*-12 plants grown on Jiffy for five weeks, displaying a partial reversal of the late-flowering phenotype. Early bolting was observed, and plant height measurements are shown to the right. (E) Specific numbers of edited plants and editing frequencies on the *FWA* promoter target windows in T_1_ plantlets with Col-0 or *fwa* epimutant background. (F) Combined analysis of editing frequencies across the three promoter targets of *FWA*, showing the total numbers of edited plants and editing frequencies for each group of editor transformants.

**Figure S6.** Flowering phenotype of the edited offspring in T_2_ and T_3_ generations. (A, B) Representative flowering phenotype of 5- (A) and 7- (B) week-old T_2_ edits for proFWA-TALEN1 in *fwa*-12 and proFWA-ngTALEN1 in *fwa*-23. (C) GFP-free seeds of the T_3_ edits for proFWA-TALEN1 in *fwa*-12-1 and proFWA-ngTALEN1 in *fwa*-23-1. GFP-positive seeds of proFWA-TALEN1 in *fwa*-12-3 were used as a control. (D) Flowering phenotype of 5-week-old T_3_ edits for proFWA-TALEN1 in *fwa*-12-1-1 and proFWA-ngTALEN1 in *fwa*-23-1-1. (E) Flowering phenotype of other 5-week-old T_2_ edits. (F) Analysis of the *cis*-elements in the promoter region and prediction of protein domains for FWA.

**Figure S7.** TALEN and ngTALEN editing of the *FWA* promoter leads to the heritable reversal of the *fwa* late-flowering phenotype in the T_3_ generation. (A) Representative flowering phenotype of 5-week-old T_3_ edited *FWA* mutants grown under long-day conditions. (B) Genotyping results of TALEN and ngTALEN in T_3_ transformants are shown, with multiplex genomic PCR data for representative edited lines. Some edited plants segregated away from the transgene and did not express *NPTII*. (C) A bar graph displays *FWA* expression levels in edited T_3_ plants. Each dot represents data for one sample per replicate. One-way ANOVA test suggested that *FWA* expression was highly upregulated in the *fwa-d* mutant but suppressed in Col-0 and lines of proFWA-ngTALEN1 in *fwa*-23, in leaves of two-week-old plants. (D) Methylation status at the target window. Filled bar, methylated. Empty bar, unmethylated. (E) Representative Sanger sequencing results illustrate the stable and heritable mutations induced by the genome editors in T_3_ edited plants. The wild-type sequences of Col-0 and *fwa* are provided at the top.

**Figure S8.** Comparison of editing efficiency of editors across various sites. (A) Violin plot illustrating the combined analysis of total editing and homozygous editing frequencies across all six target sites on *FWA* locus in T_1_ transformants with either Col-0 or *fwa* epimutant backgrounds. (B) Summary of combined analysis of editing frequencies for all six targets at the *FWA* locus, indicating the total number of edited plants and editing frequency for each group of editor transformants.

**Figure S9.** Two-sgRNA vector construction. (A) Flowchart of the two-sgRNA vector construction. Cas9 nuclease is driven by the *RPS5A* promoter, and transcription is terminated by the *HSP* terminator. The sgRNA is controlled by the *Arabidopsis U6-26* promoter. Primary transformants can be selected due to a proOle-eGFP expression cassette for floral-dipping-mediated transformation. The different domain structures are highlighted. (B) Cytosine methylation in the binding sites of MCsite4 and the ngTALEN arms using NG instead of HD to recognize 5mC. (C) Colony PCR identification of positive clones for entry vector construction. M: DNA marker. Numerical numbers represent different individual colonies. (D) PCR amplification of the whole proU6-26-CHLI2sgRNA-T35S expression cassette (left) and *Xba*I digestion of the target vector (right). (E) Two-sgRNA ligation into an entry vector *via* In-Fusion reaction. Primers binding to the two sgRNA cassettes were used for amplification. (F) Destination vector construction through multiple LR reactions. Primers covering the two sgRNA expression cassettes were used for the amplification. (G) Primer binding sites for each step of the vector construction are presented. All primers are listed in Table S1.

**Figure S10.** Comparison of editing efficiency of editors targeting the MCsite4 loci. (A) Schematic of targeting sites, highlighting the editing window, sgRNA binding sites, PAM sequence (bold, underlined), and paired TALEN binding sites for MCsite4 (multicopy CRISPR site 4). CRISPR/Cas9, TALEN, and ngTALEN share an overlapping editing window. The MCsite4.1 locus in Col-0 is depicted as an example. Red base C signifies hypermethylation. The editing window is enclosed in a rectangle. (B) Quantitative PCR for genome editors targeting MCsite4, using *PEX4* as the internal control. Twenty individual plants for each construct were sampled independently, with each sample having three technical replicates. Each dot represents data for one replicate per sample. One-way ANOVA testing indicated that the expression of TALEN and ngTALEN was significantly lower than that of CRISPR/Cas9 in leaves of two-week-old edited plants. (C) Combined analysis of editing frequencies for the four targets of MCsite4, indicating the total numbers of edited plants and editing frequency for each group of the editor transformants.

**Figure S11.** Comparison of editing efficiency of editors targeting the MCsite5 loci. (A) DNA methylation levels across the MCsite5 target sites in Col-0. Multiple WGBS datasets for seedling, shoot, and leaf tissues at the targeting locus in Col-0 were sourced from the AraENCODE database V1.1 and re-visualized as a heatmap. Methylation levels are normalized to a color scale of 0 to 1, where 1 indicates the highest level (darkest color) and 0 indicates the lowest level (white). (B) Violin plot depicting the total (left) and homozygous (right) editing frequencies for the three targets on MCsites5 in T_1_ editor transformants. (C) Combined analysis of editing frequencies for the three targets of MCsite5, indicating the total numbers of edited plants and editing frequency for each group of editor transformants. In the violin plots, each data point represents the editing frequency for a set of 10 plants, representing a replicate for a specific editor. Abbreviations: homo, homozygous editing; h/c, heterozygous/chimera editing. The middle-dashed line signifies the means. The other dashed lines represent the interquartile ranges.

**Figure S12.** Chromatin states at the *FWA* locus in Col-0 and *met1-3* plants. (A) Chromatin states at the *FWA* locus in Col-0 plants. (B) Chromatin states at the *FWA* locus in *met1-3* mutant plants. The figure was generated by accessing AraENCODE database (Wang et al., 2023) on November 8, 2025.

**Table S1. alphafold3 modelling of the overall interaction**

| Editor protein | Target DNA | pTM | ipTM |
| --- | --- | --- | --- |
| MCsite4-TALEN-left | AGCCGAGACCAGTTTTC | 0.72 | 0.73 |
| MCsite4-TALEN-right | TATATTTCTGTCCGAACCA | 0.72 | 0.82 |
| MCsite4-TALEN-left | AG**CC**GAGAC**C**AGTTTTC | 0.69 | 0.77 |
| MCsite4-TALEN-right | TATATTT**C**TGT**CC**GAAC**C**A | 0.65 | 0.75 |
| MCsite4-ngTALEN-left | AGCCGAGACCAGTTTTC | 0.7 | 0.7 |
| MCsite4-ngTALEN-right | TATATTTCTGTCCGAACCA | 0.68 | 0.65 |
| MCsite4-ngTALEN-left | AG**CC**GAGAC**C**AGTTTTC | 0.73 | 0.88 |
| MCsite4-ngTALEN-right | TATATTT**C**TGT**CC**GAAC**C**A | 0.68 | 0.83 |
| MCsite4-CRISPR/Cas9 | GTTTTCTCGCACTTAAGCTCTGG | 0.91 | 0.88 |
| MCsite4-CRISPR/Cas9 | GTTTTCT**C**GCA**C**TTAAGCT**C**TGG | 0.92 | 0.89 |

Note: Methylated cytosine is labeled in red. pTM represents the predicted template modeling score, while ipTM refers to the interface predicted template modeling score.

**Table S2. Primers used**

| **sgRNAs** | | |
| --- | --- | --- |
| Constructs | Primer direction | Primer sequences |
| proFWA-CRISPR1 | proFWA-sgRNA1-Fw | 5'-ATTGTTCATACGAGCGCCGCTCTA-3' |
|  | proFWA-sgRNA1-Rv | 5'-AAACTAGAGCGGCGCTCGTATGAA-3' |
| proFWA-CRISPR2 | proFWA-sgRNA2-Fw | 5'-ATTGGAAAAGCAAAAACCGTAAAG-3' |
|  | proFWA-sgRNA2-Rv | 5'-AAACCTTTACGGTTTTTGCTTTTC-3' |
| proFWA-CRISPR3 | proFWA-sgRNA3-Fw | 5'-ATTGGGTATATATGAGATTCTCGA-3' |
|  | proFWA-sgRNA3-Rv | 5'-AAACTCGAGAATCTCATATATACC-3' |
| cdsFWA-CRISPR1 | cdsFWA-sgRNA1-Fw | 5'-ATTGTGGTAGATGAAAGGGTCGAG-3' |
|  | cdsFWA-sgRNA1-Rv | 5'-AAACCTCGACCCTTTCATCTACCA-3' |
| cdsFWA-CRISPR2 | cdsFWA-sgRNA2-Fw | 5'-ATTGGGATTTTCGATAGTGCCAGA-3' |
|  | cdsFWA-sgRNA2-Rv | 5'-AAACTCTGGCACTATCGAAAATCC-3' |
| cdsFWA-CRISPR3 | cdsFWA-sgRNA3-Fw | 5'-ATTGGGCACTATCGAAAATCCCGA-3' |
|  | cdsFWA-sgRNA3-Rv | 5'-AAACTCGGGATTTTCGATAGTGCC-3' |
| MCsite4-CRISPR | MCsite4-sgRNA-Fw | 5'-ATTGGTTTTCTCGCACTTAAGCTC-3' |
|  | MCsite4-sgRNA-Rv | 5'-AAACGAGCTTAAGTGCGAGAAAAC-3' |
| MCsite5-CRISPR | MCsite5-sgRNA-Fw | 5'-TAGGTGGACTATGCAGTCAA-3' |
|  | MCsite5-sgRNA-Rv | 5'-TTGACTGCATAGTCCACCTA-3' |
| CHLI2-CRISPR | CHLI2sgRNA-Fw | 5'-ATTGCGACATTCATAACAGAGACA-3' |
|  | CHLI2sgRNA-Rv | 5'-AAACTGTCTCTGTTATGAATGTCG-3' |

| **Colony PCR primers** | | |
| --- | --- | --- |
| Vector | Primer direction | Primer sequences |
| pEnChimera | pEnChimera-Fw | 5'-GGGAATCTGAAAGAAGAGAA-3' |
| pDESTpK7WG2-pRPS5A-Cas9-NLS-pOle-eGFP | pK7WG2-NLSCas9-Fw | 5'-TCTCTCACAGCTCGGTGGTG-3' |
|  | pK7WG2-NLSCas9-Rv | 5'-CGTCGCATGCCTGCAGGTCA-3' |
| pDESTpK7WG2  -RPS5Ap-NLS-OleGFP | pDEST-TAL-Fw | 5'-TTCTCTTAGGTTTACCCGCC-3' |
|  | pDEST-TAL-Rv | 5'-CCATAGTCCATACCATAGCAC-3' |
| pEnChimera-pU6-26-CHLI2sgRNA-scaffold | infusionCHLI2-Fw | 5'-GTGCTTTTTTTCTAGGCTCTTTTTTTCTTCTTCTT-3' |
|  | infusionCHLI2-Rv | 5'-GAAAGCTGGGTCTAGAAAAA-3' |

| **Amplicon identification/sequencing** | | |
| --- | --- | --- |
| DNA fragment | Primer direction | Sequences |
| *proFWA* | sFWApro-Fw | 5'-GAATCAATTGGGTTTAGTGTTTACTTG-3' |
|  | sFWApro-Rv | 5'-CGGGAACCAAAATCATTCTCTAA-3' |
| *proFWA* | bsFWApro-Fw | 5'-AATTTAGTAAAGAATTAATTGGGTTTAGTGT-3' |
|  | bsFWApro-Rv | 5'-CGACGCAAAATCTAATATTTAACTAAAA-3' |
| *cdsFWA* | sFWAcds-Fw | 5'-CACCAACTCTACCAGCCTTTGA-3' |
|  | sFWAcds-Rv | 5'-TCTCGACACTTTTGACAGTACCTTG-3' |
| *MCsite4.1* | MCsite4.1-Fw | 5'-ACGAATGGCTTGATCCCTTC-3' |
|  | MCsite4.1-Rv | 5'-TTCTGGGCTCTGTTTCAAGAATA-3' |
| *MCsite4.3* | MCsite4.3-Fw | 5'-AATACGAACTACGAATGGCTTGA-3' |
|  | MCsite4.3-Rv | 5'-TCCAGCAGGCTTATGTATGATTT-3' |
| *MCsite4.4* | MCsite4.4-Fw | 5'-ACTGGGTACGTAGGCAATCC-3' |
|  | MCsite4.4-Rv | 5'-TAAAAGTATTCGGCGTGTACTGG-3' |
| *MCsite4.7* | MCsite4.7-Fw | 5'-AATCCGACCTCTCCGTTAGAACT-3' |
|  | MCsite4.7-Rv | 5'-AAAATAAGTGAGGCGGACTCAGCA-3' |
| *CHLI2* | CHLI2-Fw | 5'-GTCCCATCTCTGCTTCGGAC-3' |
|  | CHLI2--Rv | 5'-CACACCACCTATCTTCGGGT-3' |
| *NPTII* | NPTII-Fw | 5'-TTGCTCCTGCCGAGAAAGTAT-3' |
|  | NPTII-Rv | 5'-GGTAGCCAACGCTATGTCCTG-3' |
| *ACTIN2* | ACTIN2-Fw | 5'-GTATGAGCAAAGAAATCACAGCACT-3' |
|  | ACTIN2-Rv | 5'-TGTGAACGATTCCTGGACCTG-3' |

| **qRT-PCR primers** | | |
| --- | --- | --- |
| DNA fragment | Primer direction | Primer sequences |
| *PEX4* | qPEX4-Fw | 5'-CTGCGACTCAGGGAATCTTCTA-3' |
|  | qPEX4-Rv | 5'-TCATCCTTTCTTAGGCATAGCG-3' |
| *CRISPR-Cas9* | qCRISPR-Fw | 5'-GAGACAAACGGTGAAACAGGTG-3' |
|  | qCRISPR-Rv | 5'-CTTAGGGTCCCAATCCTTCTTC-3' |
| *TALEN* | qTALEN-Fw | 5'-GACCCGCACTGGAGTCAATT-3' |
|  | qTALEN-Rv | 5'-GCTGGGAACGGCTGATAGG-3' |
| *FWA* | qFWA-Fw | 5'-ATAGGCGCACTGCTTATCAAAC-3' |
|  | qFWA-Rv | 5'-CTTCTAAGCTGATCCTGAGTTGCT-3' |

**Reference:**

Nakazato, I., Okuno, M., Yamamoto, H., Tamura, Y., Itoh, T., Shikanai, T., Takanashi, H., Tsutsumi, N. and Arimura, S.-i. (2021) Targeted base editing in the plastid genome of *Arabidopsis thaliana*. *Nat Plants* **7**, 906-913.

Wang, Z., Liu, M., Lai, F., Fu, Q., Xie, L., Fang, Y., Zhou, Q., and Li, G. AraENCODE: A comprehensive epigenetic database of *Arabidopsis thaliana*. *Mol Plant* **16**, 1113-1116.
